# Evaluation of Clazakizumab (Anti–Interleukin-6) in Patients With Treatment-Resistant Chronic Active Antibody-Mediated Rejection of Kidney Allografts

**DOI:** 10.1016/j.ekir.2022.01.1074

**Published:** 2022-02-09

**Authors:** Stanley C. Jordan, Noriko Ammerman, Jua Choi, Edmund Huang, Reiad Najjar, Alice Peng, Supreet Sethi, Rana Sandhu, Janet Atienza, Mieko Toyoda, Shili Ge, Kathlyn Lim, Matthew Gillespie, Xiaohai Zhang, Mark Haas, Ashley Vo

**Affiliations:** 1 Transplant Immunotherapy Program, Comprehensive Transplant Center, Cedars-Sinai Medical Center, Los Angeles, California, USA

**Keywords:** antibody-mediated rejection, anti–IL-6, donor-specific antibodies, HLA-incompatible transplantation

## Abstract

**Introduction:**

Interleukin-6 (IL-6) is an important mediator of inflammation and activation of T cells, B cells, and plasma cells. Excessive IL-6 production is linked to human diseases characterized by unregulated antibody production, including alloimmunity, where persistence of donor-specific antibodies (DSAs), chronic active antibody-mediated rejection (cAMR), and graft loss are noted. Here, we report our experience investigating clazakizumab, a novel IL-6 inhibitor, in treating human leukocyte antigen (HLA)-sensitized patients with cAMR.

**Methods:**

Between February 2018 and January 2019, 10 adults with biopsy-proven cAMR were enrolled in a phase 2, single-center, open-label study. Patients received clazakizumab 25 mg subcutaneously (s.c.) monthly for 12 months, with a 6-month protocol biopsy. Primary end points included patient survival, graft survival, estimated glomerular filtration rate (eGFR), and safety. Secondary end points assessed immune markers (DSAs, IgG, T-regulatory [Treg] cells). At 12 months, stable patients entered a long-term extension (LTE).

**Results:**

LTE patients received clazakizumab for >2.5 years. Mean eGFRs showed significant declines from −24 months to study initiation (0 months) (52.8 ± 14.6 to 38.11 ± 12.23 ml/min per 1.73 m^2^, *P* = 0.03). However, after initiation of clazakizumab, eGFR stabilized at (41.6 ± 14.2 and 38.1 ± 20.3 ml/min per 1.73 m^2^, at 12 and 24 months, respectively). Banff 2017 analysis of pre- and post-treatment biopsies showed reductions in g+ptc and C4d scores. DSA reductions were seen in most patients. Adverse events (AEs) were minimal, and 2 graft losses occurred, both in patients who discontinued clazakizumab therapy at 6 months and 12 months after study initiation.

**Conclusion:**

In this small cohort of patients with cAMR, clazakizumab treatment showed a trend toward stabilization of eGFR and reductions in DSA and graft inflammation. No significant safety issues were observed. A randomized, placebo-controlled clinical trial (IMAGINE) of clazakizumab in cAMR treatment is underway (NCT03744910).

## Graphical abstract

**Figure:**
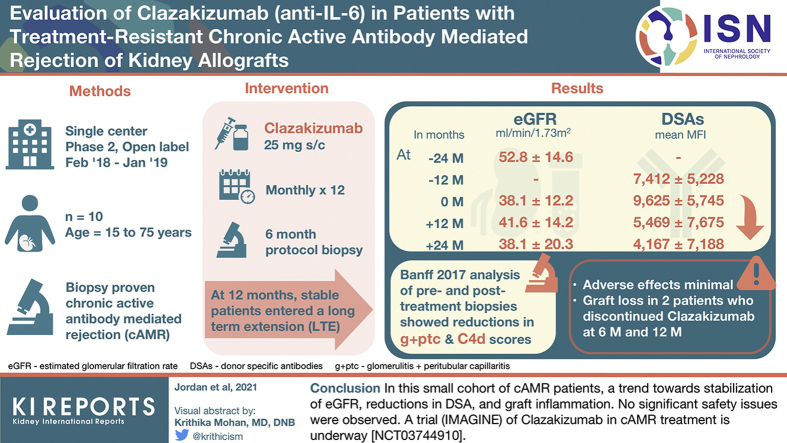

See Commentary on Page 678

Although improvements in 1-year kidney allograft survival rates are now seen, the most disappointing aspect of kidney transplantation is the lack of improvement in long-term survival. This is despite improvements in clinical care and advances in immunosuppression. The driving force behind declining graft survival is development of allosensitization to HLA and other immunogenic targets in the allograft. Here, sensitization to HLA antigens results in immune cell activation, resulting in development of DSAs that mediate acute and chronic injury to allografts. DSAs develop in ∼30% patients by 10 years after transplant. When considered in the context of other pathologic events, it is not surprising that cAMR is responsible for the majority of graft losses.^1^, ^2^, ^3^ In another study, investigators reported that cAMR was responsible for graft injury and loss in 60% of renal allografts analyzed. Our understanding of the immunopathology and immune mechanisms of injury mediated by alloantibodies has grown enormously. However, efforts to treat cAMR have not been effective. A consensus conference on recommending treatments for cAMR reported that the aims of treatment should be to preserve renal function, reduce histologic injury, and reduce DSA levels; however, the assessments of current therapies showed that “there was no conclusive evidence to support any specific therapy.”^4^ The working group also concluded that “properly conducted and powered clinical trials of biologically plausible agents are urgently needed to improve patient outcomes.”^4^

IL-6 is a pleiotropic, multifunctional cytokine that could represent an important target for modification of AMR and cAMR.^5^ IL-6 is important in activation of T follicular helper (Tfh) and T helper 17 cells, plasmablast and plasma cells. Data from animal models have shown that anti–IL-6/IL-6 receptor (IL-6R) treatment can reduce T helper 17 and Tfh activation, reduce plasmablast and plasma cell IgG production, and induce Treg cells.^5^ Data from transplant recipients have shown IL-6 as a proinflammatory cytokine associated with AMR. Reports have shown that kidney transplant recipients frequently exhibit elevated serum and urinary IL-6 levels after transplantation and during rejection episodes.^6^^,^^7^ IL-6 mRNA transcripts have also been identified in renal biopsies from patients with acute rejection.^8^

Our group examined IL-6 serum concentrations in HLA sensitized (HS) patients after kidney transplant, in comparison over time with those without graft injury, AMR, cell mediated rejection, and ischemic injury. We observed significant increases in serum IL-6 before rejection in HS patients that was not observed in patients without rejection.^5^

Since Food and Drug Administration approval, we have investigated tocilizumab (anti–IL-6R) therapy in the treatment of cAMR. Patients with cAMR treated with long-term tocilizumab therapy showed a significant benefit in graft survival, DSA reduction, and eGFR stabilization over a 6-year observation period compared with a historical cohort of patients with cAMR (*n* = 39) receiving i.v. immunoglobulin + rituximab. Patients received 6 to 12 months of tocilizumab 8 mg/kg IV *(n* = 37).^9^ Pre-tocilizumab and post-tocilizumab treatment biopsies available in selected patients revealed improvements in some features of AMR, including reduced C4d+ scores and inflammatory infiltrates in the glomeruli and peritubular capillaries. Similar observations have been reported by others.^5^^,^^10^^,^^11^

On the basis of this observed benefit of IL-6 inhibition in treating cAMR, we began an investigator-initiated trial of a novel monoclonal antibody, clazakizumab (anti–IL-6), for cAMR treatment in February 2018 (Clinicaltrials.gov NCT03380377).^12^

Clazakizumab (CSL Behring, LLC, King of Prussia, PA) is a genetically engineered humanized IgG1 antibody that binds with high potency to human IL-6. The clazakizumab development program includes a comprehensive nonclinical development approach with completed clinical studies conducted in healthy subjects and in subjects with rheumatoid arthritis, psoriatic arthritis, Crohn disease, graft-versus-host disease, and oncology indications. Clazakizumab is not yet Food and Drug Administration approved for any condition.

## Methods

### Study Design

**Figure 1:**
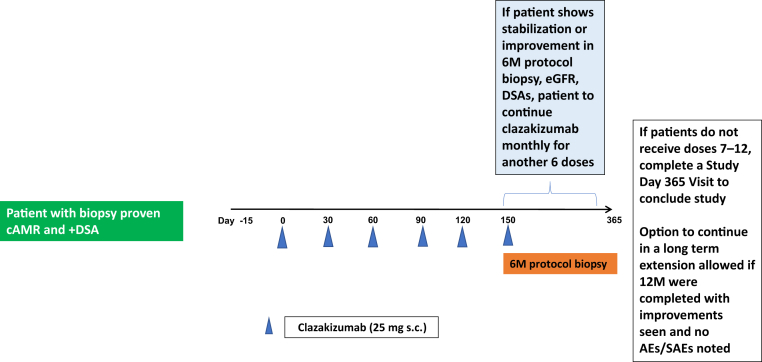
This figure shows the protocol design. Patients eligible for the study were DSA positive with clinical features and pathologic features of cAMR. All patients received clazakizumab 25 mg s.c. monthly for 6M followed by protocol biopsy. If improvements were seen, patients received clazakizumab monthly for an additional 6M with the option to continue in a LTE if improvements were seen and no AEs/serious AEs noted. AE, adverse event; cAMR, chronic active antibody-mediated rejection; DSA, donor-specific antibody; eGFR, estimated glomerular filtration rate; LTE, long-term extension; M, months; s.c., subcutaneously; SAE, serious adverse event.

This study was a phase 2, open-label, single-arm investigator-initiated trial (Stanley Jordan, MD, principal investigator) developed, conducted, and evaluated by the investigators after approval from Vitaeris, Inc. and CSL Behring, LLC. The study was designed and approved to enroll a single cohort of 10 kidney transplant patients with cAMR with monitoring and follow-up for 12 months. The study has been conducted in accordance with the Declaration of Helsinki, with the ethics guidelines based on federal regulations and the Common Rule. It was approved by the Institutional Review Board at Cedars-Sinai Medical Center (CSMC), in Los Angeles, California. All patients were recruited from the CSMC Kidney Transplant Program, and all study activities occurred at CSMC, after enrolled subjects provided written consent for participation in the trial. Ten patients were enrolled between February 2018 and January 2019. The study design is depicted in Figure 1.

### Enrollment

Eligibility criteria included patients aged 15 to 75 years old with biopsy-proven cAMR as defined by Banff criteria 2017 and DSA + at time of biopsy, who were able to understand and provide informed consent, were pneumococcal vaccinated, and with a negative tuberculosis test. Key exclusion criteria were as follows: multiorgan transplant; eGFR <30 ml/min per 1.73 m^2^; advanced TG (CG3) or advanced IFTA (ct or ci scores ≥3); previous allergic reactions to monoclonal antibodies; lactating or pregnant females; women of child-bearing age and male partners of women of child-bearing age who are not willing or able to practice Food and Drug Administration-approved forms of contraception; HIV, hepatitis B virus, or hepatitis C virus positive patients; those with latent or active tuberculosis; recent recipients of any live attenuated vaccine(s) 2 months before the screening visit; abnormal laboratory results defined as white blood cell <3.0 × 10^3^/ml, hemoglobin <8.0 g/dl, platelet count <100 × 10^3^/ml, and aspartate transaminase or alanine transaminase >3× the upper limit normal; individuals deemed unable to comply with the protocol; subjects with active cytomegalovirus or Epstein-Barr virus infection; use of investigational agents within 4 weeks; history of or active inflammatory bowel disease; diverticular disease or gastrointestinal perforation; recent infection (within past 6 weeks of screening) requiring any antibiotic use; and present or previous (within 5 years) malignancy except for basal cell carcinoma, fully excised squamous cell carcinoma of the skin, or nonrecurrent cervical carcinoma-in-situ. The full protocol and related documents are included in the Supplementary Material digital content. No pediatric patients were entered into this study.

### Study Treatment

A total of 9 of 10 enrolled patients received clazakizumab 25 mg s.c. injections monthly for 12 doses, whereas 1 discontinued at 6 months after study initiation. Patients underwent a 6-month protocol biopsy. For the first 6 months of the study, monitoring of renal function, DSAs, and Banff 2017 biopsy scores were evaluated. If improvement or stabilization of clinical features for cAMR and biopsy were observed in comparison to baseline, patients were able to receive the final 6 monthly clazakizumab doses. In the event a patient did not show improvement after receiving the initial 6 doses of clazakizumab, no further treatment would be administered, and the patient would return at day 365 for a final study visit. After 12 months of therapy, assessment of patient and graft survival, stabilization of clinical features of cAMR (eGFR, serum creatinine), and safety throughout study duration were assessed. At study cessation, patients were provided the option to enter into a LTE to receive clazakizumab 25 mg s.c. every other month. LTE patients were reviewed to assess potential benefit from study treatment and continued monitoring for safety and AEs at each patient appointment.

### Study Objectives

Because of the small sample size and single-treatment arm design, we did not expect to obtain statistically significant assessments of efficacy from this study. The primary objective was to assess the safety and limited efficacy of clazakizumab in eliminating DSAs and stabilizing clinical features of cAMR in patients who have biopsy-proven severe cAMR. Safety determinations included assessments of any side effects associated with clazakizumab administration and risk for infectious complications and gastrointestinal issues. Owing to the small sample size, only limited efficacy determinations included (i) assessment of patient and graft survival, (ii) stabilization of the clinical features of cAMR (eGFR, serum creatinine) after clazakizumab treatment, and (iii) safety throughout study duration.

Secondary end points were (i) allograft function up to 6 to 12 months, (ii) renal function, determined using serum creatinine, (iii) MDRD eGFR (Schwartz equation for patients under 18 years of age) calculations, and (iv) DSA levels. A protocol biopsy was performed at 6 months after clazakizumab therapy. Any late rejection episodes after clazakizumab therapy would be recorded, and several immunologic determinations were assessed at time points before and after initiation of clazakizumab therapy (baseline, 3 months, 6 months, and 12 months).

Enrolled patients continued on their existing maintenance immunosuppression regimens with tacrolimus or cyclosporin, everolimus or sirolimus, mycophenolate mofetil, mycophenolic acid or azathioprine, and corticosteroids. Dose adjustments were made according to standard practice. Throughout the first year of the study protocol, Epstein-Barr virus, cytomegalovirus, and polyoma BK DNA polymerase chain reactions were collected and reviewed quarterly (baseline, 3 months, 6 months, 9 months, 12 months). During the LTE protocol, viral monitoring occurred twice a year (weeks 24 and 52). Infection prophylaxis was not included in the study protocol. Infectious complications throughout therapy were closely monitored, and dose interruptions were planned if an infection occurred. Clazakizumab dose interruptions would occur with an absolute neutrophil count 500 to 1000 cells/mm^3^, platelet count 50 to 100,000 cells/mm^3^, or an aspartate transaminase or alanine transaminase >3 to 5× upper limit normal. Protocol kidney allograft biopsies were performed at 6 months. Specimens were analyzed according to Banff 2017 classification. Treatment of acute rejection episodes were per center protocol.

### Donor-Specific Anti–HLA Antibody Testing

HLA antibodies were detected by the single antigen bead-based assay (One lambda, Los Angeles, CA). Donor HLA–specific antibodies were assessed throughout clazakizumab treatment for the first year and biannually during the LTE. Detection of DSAs were only reported with MFI >2500. All samples collected during the study period were treated with EDTA to improve accuracy of DSA detection and eliminate nonspecific binding to assay beads. All samples were analyzed at the CSMC HLA and Immunogenetics Laboratory (ASHI certified) using Luminex testing platforms.

### Treg Cell Flow Cytometry Analysis

Treg cell flow cytometry analysis was performed at CSMC Transplantation & Immunology Laboratory. Briefly, sodium heparinized peripheral blood were first stained with antibodies to CD45 (Horizon V500, BD Biosciences, San Jose, CA), CD3 (FITC, Invitrogen), CD4 (PerCP-Cy5.5, BD Biosciences), CD25 (Horizon V450, BD Biosciences), and CD127 (APC, eBioscience). After lysing red blood cells followed by permeabilization, the cells were stained with antibody to Foxp3 (PE, BD Biosciences). After acquiring the cell by flow cytometry, lymphocyte separated from CD45+ leukocytes were plotted against CD4. CD4+ cells were plotted as CD25 versus CD127 and then CD25+CD127 low/− cells against Foxp3. CD4+/CD25+/CD127 low/−/Foxp3+ cells were designated as Treg cells. Treg cell levels were expressed as Treg cell% in CD4+ T cells.

### Statistical Analysis

Owing to the exploratory nature of this study that involved safety end points and a small sample size that was not powered for efficacy end points, our primary objective was to assess safety end points and limited efficacy. Most importantly, we assessed tolerability and determined the effects of clazakizumab treatment on circulating DSAs and the ability to improve outcomes of patients with cAMR.

## Results

### Patient Characteristics

**Figure 2:**
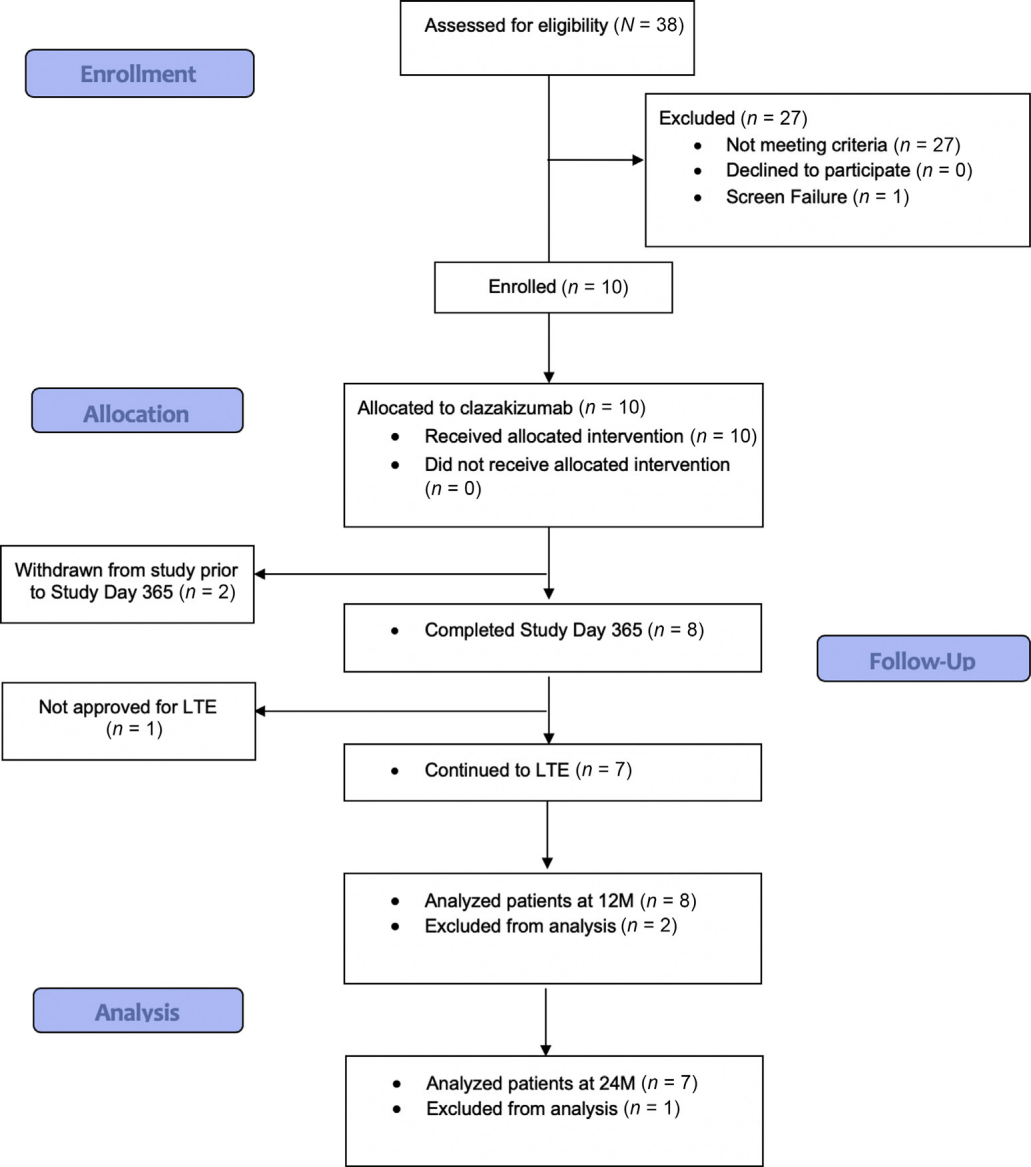
Figure 2 shows the study flow enrollment diagram. The study was approved for 10 patients with cAMR. cAMR, chronic active antibody-mediated rejection; M, months.

Between February 2018 and January 2019, a total of 10 patients fulfilling the inclusion criteria were enrolled into this single group, open-label study. Enrolled patients were a median of 8.2 years post-transplant. All patients received clazakizumab 25 mg s.c. injection monthly and underwent a protocol biopsy at 6 months. Median eGFR at study entry was 41.90 ± 12.09 ml/min per 1.73 m^2^. A flowchart of the patients enrolled in the study is shown in Figure 2. Baseline demographic and transplant characteristics are available in Table 1. At study enrollment, 9 of the 10 patients were on a tacrolimus-based immunosuppression regimen. All patients in this study had a tacrolimus goal trough of 4 to 6 ng/ml except the concomitant everolimus patient (tacrolimus goal trough of 2–5 ng/ml with everolimus goal trough of 3–8 ng/ml); cyclosporin goal trough was 75 to 125 ng/ml. Doses of mycophenolate mofetil, mycophenolic acid, or azathioprine were reduced for each participant, starting at the time of the first clazakizumab dose administration. All patients received prednisone 5 mg daily. Of 10 patients, 2 were withdrawn before completing the first year of study. One patient failed to comply with the study protocol and was removed from the study after 3 doses of clazakizumab. The second patient preferred to return to tocilizumab (anti–IL6-R) as cAMR therapy after receiving 7 doses of clazakizumab. One patient completed the first year of study (day 365) but was not approved to proceed to the LTE owing to progressive decline in kidney function. This patient traveled to our center from out of state for monthly clazakizumab therapy; it was determined that there was not enough benefit from treatment to justify travel for the LTE study. However, both patients were followed up for the duration of evaluation (+24 months), and data were analyzed for 9 of 10 patients originally entered into the study. The remaining 7 of the 10 enrolled patients completed the first year of study and elected to continue to the LTE. Thus, 7 of 10 patients have now undergone 2.5 years of study participation with clazakizumab administration (see Table 2 for results).

**Table 1:** Patient and recipient characteristics at time of enrollment

Patient characteristics at enrollment	Clazakizumab group (*N* = 10)
Age, y, mean (range)	51 (21–70)
Sex, female, n (%)	3 (30)
Weight, kg, mean (range)	79 (57.2–134.3)
Prior transplant, *n* (%)	
1 prior transplant	7 (70)
>1 prior transplant	3 (30)
ESRD etiology, *n* (%)	
Glomerulonephritis	1 (10)
Congenital hypoplastic kidney	1 (10)
Hypertension	2 (20)
Medullary cystic kidney disease	1 (10)
Obstructive uropathy, posterior urethral valves with reflux	1 (10)
Granulomatosis with polyangiitis	1 (10)
Interstitial nephritis	1 (10)
Hemolytic uremic syndrome	1 (10)
Unknown	1 (10)
Donor type, *n* (%)	
Living, related	2 (20)
Living, unrelated	3 (30)
Deceased donor	5 (50)
Time since transplant, days	
Mean (range)	2997 (1126–8113)
DSA MFI, mean (SD)	9625 (5745)
DSA, *n* (%)	DQ, 8 (80) DR, 1 (10) DP, 1 (10)
Induction agent, *n* (%)	
Basiliximab	2 (20)
Alemtuzumab	5 (50)
Daclizumab	1 (10)
Unknown	2 (20)
Maintenance immunosuppression, *n* (%)	
Tacrolimus, MMF, steroid	7 (70)
Tacrolimus, AZA, steroid	1 (10)
Tacrolimus, everolimus, steroid	1 (10)
Cyclosporine, MMF, steroid	1 (10)
Tacrolimus level at study entry, mean in ng/ml (range)	4.8 (3.6–6.6)
CSA level at study entry, mean in ng/ml( *n* = 1)	97
eGFR, ml/min per 1.73 m^2^, mean (SD)	41.90 ± 12.09
Creatinine, mg/dl, mean (SD)	1.74 (0.55)
C-reactive protein, mg/l, mean (SD)	1.1 (1.25)
IgG, mg/dl, mean (SD)	809.63 (121.42)
T-regulatory cells, % in CD4+ T cells, mean (SD)	2.74 (1.09)
Past therapies received as AMR treatment, *n* (%)	
Rituximab with IVIg x1	1 (10)
Rituximab with IVIg x2	2 (20)
Rituximab with IVIg x3	1 (10)
Obinutuzumab with IVIg ×1 and tocilizumab ×6M	1 (10)
Rituximab with IVIg ×1 and tocilizumab ×6M	1 (10)
Rituximab with IVIg ×1, tocilizumab ×6M and obinutuzumab with IVIg ×1	2 (20)
Not previously treated	2 (20)

AZA, azathioprine; CSA, cyclosporin; DSA, donor-specific antibody; eGFR, estimated glomerular function rate; ESRD, end-stage renal disease; IQR, interquartile range; IVIg, i.v. immunoglobulin; M, months; MFI, mean fluorescent intensity; MMF, mycophenolate mofetil.

**Table 2:** Results

Results summary	Clazakizumab group
Patients in IIT analysis, *n*	8 (12M) 7 (24M)
DSA MFI, mean (SD)	
12M	5,469 (7,675)
24M	4,167 (7,188)
DSA, *n* (%)	
12M	DQ, 4 (50)
24M	DQ, 2 (29)
eGFR, ml/min per 1.73 m^2^, mean (SD)	
12M	36.38 (11.92)
24M	39.43 (12.25)
Creatinine, mg/dl, mean (SD)	
12M	1.93 (0.53)
24M	1.91 (0.54)
C-reactive protein, mg/l, mean (SD)	
12M	0.42 (0.14)
24M	0.53 (0.24)
IgG, mg/dl, mean (SD)	
6M	689.67 (206.95)
12M	664 (77.63)
24M	669 (182.12)
T-regulatory cells 24M, % in CD4+ T cells, mean (SD)	3.32 (1.04)

DSA, donor-specific antibody; eGFR, estimated glomerular function rate; IIT, investigator-initiated trial; M, months; MFI, mean fluorescent intensity.

**Figure 3:**
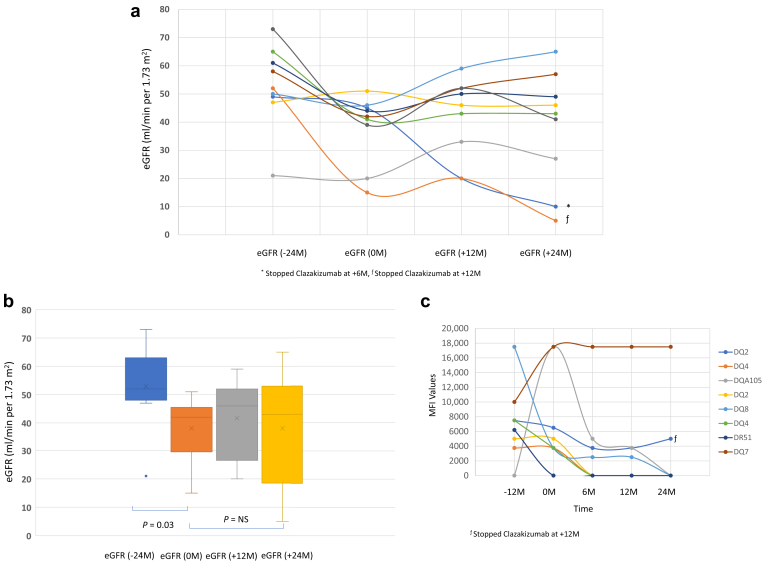
(a) Shows the individual patient curves of eGFR at study entry and at time points up to 24M after clazakizumab therapy. (b) Shows the mean eGFR at study entry and at time points up to 24M after clazakizumab therapy. Here, a stabilization of eGFR was seen over the 24-month observation. (c) This figure shows the impact of clazakizumab on DSA levels over the 2-year study period. Each line represents a patient, and each patient had only 1 DSA present from study entry and throughout study (*n* = 8). The DSAs were analyzed at the CSMC HLA Laboratory. Near significant reductions in DSAs were seen at 12 and 24M. CSMC, Cedars-Sinai Medical Center; DQ, human leukocyte antigen – DQ isotype; DR, human leukocyte antigen – DR isotype; DSA, donor-specific antibody; eGFR, estimated glomerular filtration rate; HLA, human leukocyte antigen; M, months; MFI, mean fluorescent intensity; NS, nonsignificant.

Figure 3a and b show the individual trends (Figure 3a) and mean (Figure 3b) eGFR values from −24 months, 0 months, +12 months, and +24 months for clazakizumab administration. Briefly, mean eGFRs showed significant declines from -24 months to study initiation (0 months) (52.8 ± 14.6 to >38.11 ± 12.23 ml/min 1.73 m^2^, *P* = 0.03). However, after initiation of clazakizumab eGFR stabilized at 41.6 ± 14.2 and 38.1 ± 20.3 ml/min per 1.73 m^2^ at +12 months and +24 months, respectively. The 2 patients denoted by (∗ and ƒ) in Figure 3a showing rapid declines in eGFR were the 2 patients who discontinued clazakizumab at 6 months and 12 months after study initiation, respectively. All patients had DSAs at study entry; 100% of patients’ DSAs were class II (DQ, 80%). DSAs were determined at −12, 0, +12, and +24 months of clazakizumab treatment. At −12 months, mean ± SD values were 7412 ± 5228 MFI (range: 0–18,000 MFI). At 0 months, 9625 ± 5745 *(n =* 10). At +12 months of clazakizumab therapy, mean DSA (peak MFI) was reduced to 5469±7675 (*P* = 0.21; N=8) and at +24 months to 4167±7188 (*P* = 0.12; N=7); (Table 2). Figure 3c shows the impact of clazakizumab on DSA levels for individual patients before study entry and throughout therapy. Although numerical reductions were seen, these were not significant.

**Figure 4:**
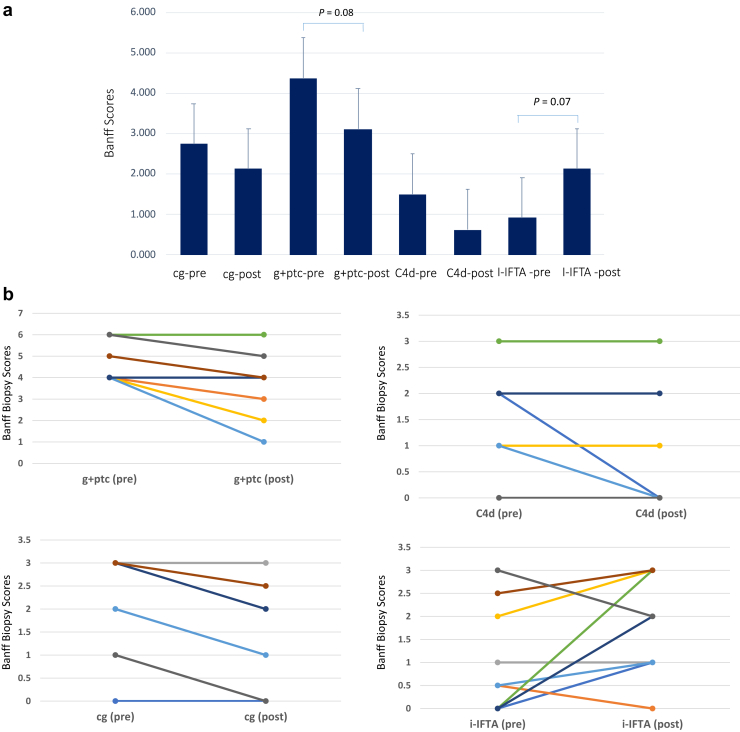
(a) This figure shows the mean Banff scores for protocol biopsies performed during the study. Near significant reductions were seen for g+ptc scores, and near significant increase in i-IFTA scores were seen after 6 months of clazakizumab therapy. (b) Shows the individual Banff scores for each patient before and after treatment.

**Figure 5:**
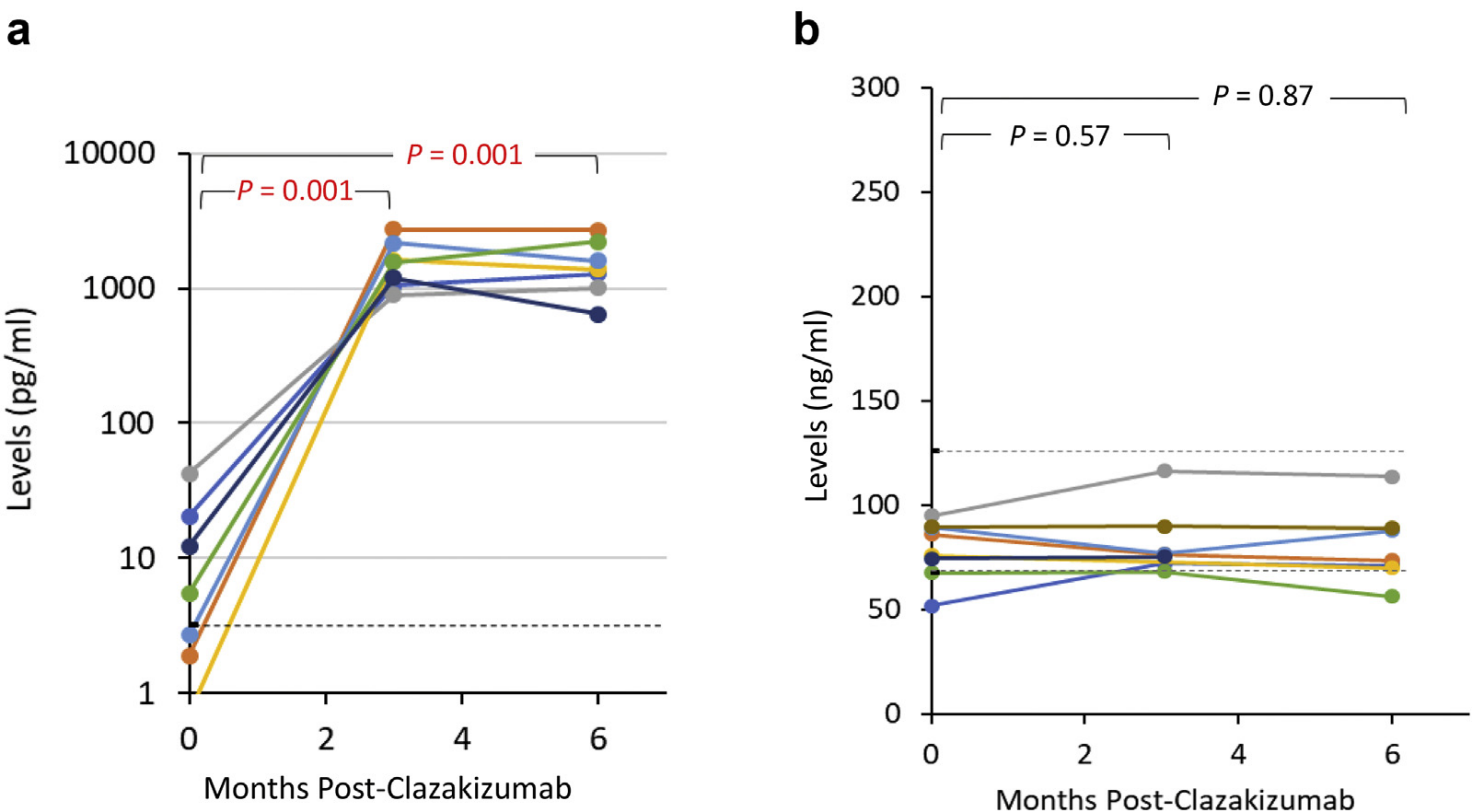
(a) Figure 5a shows pre- and postserum IL-6 levels in patients receiving clazakizumab for treatment of cAMR. The dramatic increase in serum IL-6 levels are due to stabilized IL-6/anti–IL-6 complexes in the blood. Because clazakizumab blocks the IL-6R binding site, no activation signals are possible, as was indicated with low C-reactive protein levels in all patients. (b) shows soluble IL-6R levels in the blood of patients throughout the study. Here, there are no increases in soluble IL-6R as would be seen with tocilizumab (anti–IL-6R). This may be beneficial because circulating IL-6/IL-6R complexes (called super IL-6) could form and cause severe injury at multiple tissue sites. IL-6, interleukin-6; IL-6R, interleukin-6 receptor.

**Figure 6:**
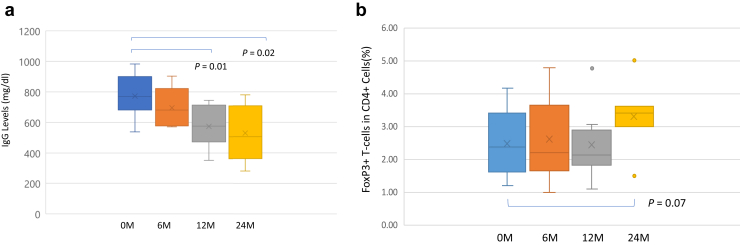
(a) This figure demonstrates the impact of clazakizumab on total IgG levels measured at initiation and every 6M up to 2 years after clazakizumab treatment. Significant decreases in total IgG levels were seen, but levels still remained in the normal range for most patients. (b) This figure shows the impact of clazakizumab treatment on CD4+, CD25+, FoxP3+ Tregs determined before treatment and at 6-month intervals up to 24M after clazakizumab treatment. Here, near significant increases in Treg cells were seen at 24M after clazakizumab treatment. M, months; Tregs, T regulatory.

All patients who completed the first 6 months of therapy underwent the 6-month protocol biopsy *(n* = 9). Six-month biopsies exhibited the following changes from the pretreatment levels in Banff 2017 scoring (Figure 4a): g+ptc 4.38 to 3.25 (*P* = 0.080), cg 2.38 to 2.00 (*P* = 0.518), v 0 to 0, C4d 1.26 to 1.00 (*P* = 0.678), and i-IFTA 0.813 to 1.75 (*P* = 0.07). Individual Banff scores for each patient are shown in Figure 4b. All patients showed increases in IL-6 levels after initiation of clazakizumab therapy. However, there were no increases in serum IL-6R levels as would be seen with tocilizumab therapy (Figure 5a and b). Here, it is important to understand that the high IL-6 levels are likely due to circulating IL-6/clazakizumab complexes and are not signaling through the IL-6R pathways because C-reactive protein levels remained <5 mg/l (Normal 0–5 mg/l) for all patients for the duration of the study. Because clazakizumab blocks the IL-6R binding site, no activation signals are possible, as was indicated with low C-reactive protein levels in all patients. Figure 5b shows soluble IL-6R levels in the blood of patients throughout the study. Here, there are no increases in soluble IL-6R as would be seen with tocilizumab (anti–IL-6R). This may be beneficial because circulating IL-6/IL-6R complexes (called super IL-6) could form and cause severe injury at multiple tissue sites. This observation has been previously described.^13^, ^14^, ^15^ Total IgG levels showed significant reductions over 24 months of therapy but remained in normal range for most patients (Figure 6a). CD4+, CD25+, CD127^low^FoxP3+ (Treg) cells showed near significant increases at 24 months (3.32% ± 1.04% vs. 2.74% ± 1.09%, *P* = 0.07; N=7; Figure 6b).

### Safety

Clazakizumab monthly and bimonthly doses were generally well tolerated without reported safety issues. Clazakizumab treatment was not halted for any participant owing to drug-related concerns. Data for serious AEs and AEs that occurred throughout therapy were recorded at each study appointment (see Table 3). Fourteen infections occurred in the study population, which accounted for 42% of the AEs reported. Two serious AEs occurred in 2 participants, who were both hospitalized for sepsis because of pyelonephritis. One participant developed Epstein-Barr virus viremia without clinical symptoms (maximum of 1736 copies/ml) after 18 months of clazakizumab therapy, which was reversed by a course of oral valacyclovir. The most common AEs during a 2.5-year period of clazakizumab administration were respiratory infection, pyelonephritis, and edema. Thrombocytopenia and hepatic enzyme elevations that can be observed with IL-6 inhibitors were not seen in our small patient population (*n* = 10). AEs observed during the initial study or the LTEs that were potentially related to treatment included pyelonephritis and upper respiratory infections. However, patients experiencing pyelonephritis had evidence for recurrent urinary infections before initiation of clazakizumab. The upper respiratory infections were seasonal and did not require hospitalizations. No gastrointestinal perorations were seen in our small study group.

**Table 3:** AEs

AE summary	# of events (*n*)
AE	33 (10)
SAE	2 (2)
Infections	
BK Infection	0 (0)
CMV Infection	0 (0)
EBV Viremia	1 (1)
Herpes Zoster Infection	2 (2)
Sepsis due to Pyelonephritis	2 (2)
Pyelonephritis	4 (3)
Respiratory Infection	3 (3)
*C. difficile* infection	1 (1)
Influenza	1 (1)
Infections by organ site, # of events (% of infections)	
Blood/systemic infection	3 (21%)
Renal and urinary disorders	4 (28%)
Respiratory disorders	4 (28%)
Skin disorders	2 (14%)
Gastrointestinal disorders	1 (7%)
Other AE	
Cough	1 (1)
Cold symptoms	1 (1)
Abdominal pain	1 (1)
Fever	1 (1)
Arm swelling/cellulitis	2 (2)
Eczema/warts on hands	1 (1)
Intermittent dizziness	1 (1)
Edema	5 (3)
Itching on legs	1 (1)
Swollen hand after exercise	1 (1)
Uric acid elevated/gout in big toe	1 (1)
Excision of skin cancer lesion	2 (2)
Anemia	3 (2)
Alopecia	1 (1)
Vaginitis	1 (1)

#, number; AE, adverse event; CMV, cytomegalovirus; EBV, Epstein-Barr virus; SAE, serious adverse event.

## Discussion

Our original work in a mouse model of allosensitization showed that inhibition of IL-6 signaling with anti–IL-6R monoclonal inhibited alloantibody generation and resulted in increased Treg and reduced Tfh cell populations.^16^ This subsequently led to human trials of anti–IL-6R for desensitization and treatment of cAMR.^9^^,^^17^ Our experience with both animal models and human studies were encouraging, especially the study of anti–IL-6R in treatment of cAMR. Here, we saw reductions in DSAs, improvements in allograft biopsy findings, stabilization of eGFR, and, more importantly, prolonged patient and graft survival when compared with a cohort of patients with cAMR treated with standard of care. These observations let us to study clazakizumab in patients with cAMR at risk for progression to end-stage renal disease.

In this study, we found that anti–IL-6 therapy given to patients with cAMR for 1 year and continued for up to 1.5 additional years in LTE *(n* = 7) resulted in a trend toward stabilization of eGFR, reduction in DSA, and increased Treg cells in the peripheral blood. Six-month protocol biopsies showed no significant changes in Banff scores; however, a trend was seen in reductions in g+ptc and increases in i-IFTA scores.

Doberer *et al.*^18^ recently reported on a randomized, placebo-controlled, cross-over trial of clazakizumab for treatment of cAMR in Europe. The dosing was similar to that of our trial with follow-up to 12 months. The investigators found significant safety signals in 5 patients under active treatment who developed serious infectious events, and 2 developed diverticular disease complications, leading to trial withdrawal. However, in patients receiving clazakizumab, significant reductions in DSAs were seen. At 1-year protocol biopsies, 38.9% of clazakizumab-treated patients showed negative molecular AMR scores. Elimination of C4d staining and resolution of morphologic features of AMR were seen in 27.8% and 22.2% of patients, respectively. EGFR decline was significantly slower in the clazakizumab-treated patients, and, during the cross-over phase, the slope of eGFR decline for patients who were switched from placebo to clazakizumab improved and no longer differed from patients initially treated with clazakizumab.

There are a number of issues to discuss regarding this important study. First, the safety signals seen in the Doberer *et al.*^18^ study were not seen in ours despite using the same dosing schedule. This is likely due to reductions of baseline immunosuppression (i.e., mycophenolate mofetil dosing not >500 mg twice daily, mycophenolic acid 360 mg twice daily) and having an exclusion criterion for patients with a history of inflammatory bowel disease, diverticulitis, or bowel perforation, which was not done in the European study. Despite this, the findings of our study are very similar to those reported in their study, which is encouraging, given the diversity in patient populations treated. Importantly, our follow-up with continued therapy is now >2.5 years showing maintenance of eGFR stability and patient safety. It will be important to determine safety profiles in larger numbers of patients included in a placebo-controlled trial.

Another important observation from our study was the increased Treg cell populations in clazakizumab-treated patients at 12 months. This suggests that continued exposure to anti–IL-6 can result in expansion of Tregs, which may have a beneficial effect in reducing graft inflammation and possibly alloantibodies. This observation is supported by recent observations of Chandran *et al.*,^19^ who conducted a randomized controlled clinical trial of patients with subclinical graft inflammation. Patients were treated with tocilizumab (8 mg/kg IV every 4 weeks; 6 doses; *n =* 16) or no treatment (controls; *n =* 14). Tocilizumab-treated patients showed improved Banff ti-score, increased Treg frequency (7.1% ± 5.55% vs. 3.6% ± 1.7%, *P* = 0.0168), and reduced T-effector cytokine response compared with controls. The authors conclude that the treatment was relatively well tolerated, with no patient deaths or graft loss. The authors summarize that inhibition of IL-6 is a novel and promising treatment option to regulate T-cell mediated immune responses in kidney transplant recipients. Other investigators have also shown that tocilizumab treatment of patients with rheumatoid arthritis significantly increases Tregs in peripheral blood, and this correlates with improvement in clinical symptoms.^20^

IL-6 is a key cytokine in driving Tfh differentiation, B cell, plasmablast, and plasma cell differentiation and pathogenic antibody production.^5^ Papillion *et al.*^21^ recently demonstrated how IL-6 amplifies Tfh cell activity, ultimately driving both T cell and B cell immunity and inflammation. These authors found that IL-6 sustains Tfh activity by inhibiting STAT-5 pathway activation. It is known that Tfh cells produce copious amounts of IL-2 when stimulated, which would ultimately terminate germinal center Tfh activity by inducing Treg cells. However, these investigators showed that IL-6 through inhibition of STAT-5 and exaggerated signaling through STAT-3 inhibited the generation of IL-2Rβ synthesis, which prevents the Tfh cells from utilizing IL-2, thus sustaining their generation and activation of antibody-secreting cells and inflammatory T cells. Here, inhibition of IL-6 would likely terminate the effect on IL-2Rβ, increase Treg cells, and allow termination of the germinal center activation events. In our study, we did observe near significant increases in Treg cells after 12 months of clazakizumab therapy along with reductions in DSA levels and improved eGFR. This will be an important issue to investigate in larger trials of anti–IL-6/IL-6R therapies.

### Ongoing Analysis

To date, there are no effective therapies for treatment of cAMR.^4^ In this regard, clazakizumab may represent a novel approach for the treatment of cAMR that allows prolongation of allograft survival beyond what is seen with accepted standard of care approaches. This will be clarified by the outcomes of the larger, multicenter, placebo-controlled study of clazakizumab that is now underway for patients with cAMR (IMAGINE, NCT03744910).

## Conclusion

Our phase 2 trial has several limitations, including the lack of a randomized controlled design with a placebo-treated control arm, a small sample size, and variability among the participants at baseline (see Table 1). However, at this stage of drug development, safety was a primary objective, and we observed clazakizumab to be well tolerated by participants, even after a prolonged duration of therapy, up to 2.5 years. We believe that the results of our study contribute to a better understanding of IL-6 inhibition in cAMR treatment, especially the observation of a trend to stabilization of eGFR. It will also be important to determine whether the impact of clazakizumab on clinical outcomes relates to generation of anti-inflammatory Treg cells, which could reduce graft inflammation and DSA production. Ultimately, the most important determinant of efficacy will be stabilization of renal function, which could delay progression of cAMR to ESRD and hopefully add years of reasonable function to the allograft.

## Disclosure

SCJ received grant support from Hansa Medical, CSL Behring, Vitaeris, and Genentech and is a consultant for Hansa Medical, CSL Behring, and Genentech. All the other authors declared no competing interests.

## Supplementary Material

Supplementary File (PDF)

**Abbreviation definitions.**

**Full Study Protocol** **.**
