# Supplementary material for: Evaluation of Clazakizumab (Anti–Interleukin-6) in Patients With Treatment-Resistant Chronic Active Antibody-Mediated Rejection of Kidney Allografts
Source: Kidney Int Rep. 2022 Feb 9;7(4):720–31. doi: 10.1016/j.ekir.2022.01.1074 (PMC9039906; doi:10.1016/j.ekir.2022.01.1074)
Supplement: Supplementary File (PDF) [file mmc1.pdf]

Abbreviation	Description	Abbreviation	Description
AMR	Antibody mediated rejection	DSA	Donor specific HLA antibody
PLEX	Plasmapheresis	CMR	Cell-mediated rejection
cAMR	Chronic active ABMR	HS	Highly HLA sensitized
TG	Transplant glomerulopathy	eGFR	Estimated Glomerular filtration rate
subQ	Subcutaneous	IVIG	Intravenous Immunoglobulin
DSAs	Donor specific antibody	M	month
HLA	Human leukocyte antigen	US	United States of America
FDA	Food & Drug Administration	CRP	c-reactive protein
IL-6	Interleukin-6	PsA	Psoriatic arthritis
IL-6R	Interleukin-6 Receptor	PD	Pharmacodynamic
Anti-IL-6R	Anti-interluekin-6 Receptor	SCr	Serum creatinine
IgG1	Immunoglobulin G1	q	every
mg	Milligram	TCMR	T-cell mediated rejection
kg	Kilogram	IVIG	Intravenous Immunoglobulin
RA	Rheumatoid arthritis	LTE	Long term extension
IRB	Institutional Review Board	IIT	Investigator-initiated trial
GVHD	Graft versus host disease	T-reg	T-regulatory cell
IS	immunosuppression	ANC	absolute neutrophil count
CSA	Cyclosporin	ULN	Upper limit of normal
SAE	Serious adverse event	ESRD	End stage renal disease
AE	Adverse event	WBC	White blood cell count
TB	Tuberculosis		

PROTOCOL

STUDY TITLE:

A Phase I/II Trial to Evaluate the Safety and Tolerability of Clazakizumab® (Anti-IL-6 monoclonal) As an Agent to Eliminate Donor Specific HLA Antibodies (DSAs) and Improve Outcomes of Patients with Chronic & Active Antibody-Mediated Rejection (cABMR) Post-Kidney Transplantation

Study Drug

Clazakizumab (Anti-IL-6 monoclonal)

Support Provided By

CSL Behring L.L.C.
1020 First Avenue
PA 19406 King of Prussia
United States

Sponsor Investigator

Name: Stanley C. Jordan, M.D.
Institution/Organization: Cedars-Sinai Medical Center
Address: 8900 Beverly Blvd.
L.A., CA. 90048
Phone: 310-423-8282
Fax: 310-423-8208
Email: sjordan@cshs.org
NCT: NCT03380377
FDA IND: 134733

Co-Investigator(s) and Research Coordinators

Ashley Vo, Pharm.D.
Director, Transplant Immunotherapy Program
Institution/Organization: Cedars-Sinai Medical Center
Address: 8900 Beverly Blvd.
L.A., CA. 90048
Phone: 310-423-2641

Nori Ammerman, PharmD
8900 Beverly Blvd. L.A., CA.90048
Phone: 310-248-8186

Coordinating Institution

Cedars-Sinai Medical Center
8700 Beverly Blvd., L.A., CA. 90048

Version 8.0
October 16, 2020

Abbreviations

Abbreviation	Description	Abbreviation	Description
ABMR	Antibody mediated rejection	SAE	Serious adverse events
PLEX	Plasmapheresis	LFT	Liver function test
cABMR	Chronic active ABMR	4W	4 weeks
TG	Transplant glomerulopathy	eGFR	Estimated Glomerular filtration rate
DeKAF	Deterioration in Kidney Allograft Function (DeKAF)	ELISA	Enzyme linked immunosorbtion assay
C4d	Complement-4d protein	PCRs	Polymerase chain reaction
DSAs	Donor specific antibody	EBV	Epstein barr virus
HLA	Human leukocyte antigen	CMV	Cytomegalovirus
FDA	Food & Drug Administration	BKV	Polyoma BK virus
IL-6	Interleukin-6	CG3	Chronic glomerulitis level 3 (severe)
IL-6R	Interleukin-6 Receptor	HBV	Hepatitis B virus
Anti-IL-6R	Anti-interluekin-6 Receptor	HCV	Hepatitis C virus
T_{reg}	T-regulatory cells	RIBA	Recombinant immunoblot assay
T_{fh}	T-follicular cells	TB	Tuberculosis
IgG	Immunoglobulin	WBC	White blood cell
dnDSA	De novo DSA	Hgb	Hemoglobin
SLE	Systemic lupus erythematosus	SGOT	Serum glutamic oxaloacetic transaminase
IgA	Immunoglobulin A	SGPT	Serum glutamic-pyruvic transaminase
CTLA4-Ig	Cytotoxic T-lymphocyte-assocated protein 4	PTLD	Post-transplant lymphoproliferative disorder
Th17	T-helper type 17	PML	Progressive Multifocal Leukoencephalopathy
IL-21	Interleukin 21	DES	Desensitization
mMR16-1	Mousenized rat- anti-mouse IL-6 receptor monoclonal antibody	AEs	Adverse events
IgM	Immunologublin M	JC	Polyoma JC virus
BM	Bone marrow	IV	Intravenous
GVHD	Graft versus host disease	EXP	Expiry date
RA	Rhuematoid arthritis	TNF	Tumor necrosis factor
CXCR5	Chemokine receptor type 5	ULN	Upper limit of normal
NHP	Non-human primate	LDL	Low density lipoprotein
MMF	Mycophenolate mofetil	CD	Crohn's disease
ATG	Anti-thymocyte globulin	MTX	Methotrexate
SOC	Standard of care	GI	Gastrointestinal
DSA+	Donor specific antibody positive	mAbs	Monoclonal antibodies
DSA-	Donor specific antibody negative		
IVIG	Intravenous Immunoglobulin		
SQ/SC	Subcutaneous		
CDC	Complement dependent cytotoxicity		
CNI	Calcineurin inhibitors		
AR	Acute rejection		
AMR	Antibody mediated rejection		
CMR	Cell mediated rejection		
sCR	Serums creatinine		
IL-10	Interleukin -10		
IL-17a	Interleukin-17a		
IFNγ	Interferon gamma		
C1q	Complement 1q protein		
HLAi	Human leukocyte antigen incompatible		
IgG1	Humanized immunoglobulin G1 antibody		
pM	Picomolar		
sIL-6R	Soluble interleukin 6 receptor		
STAT3	Signal transducer and activator of transcription 3		
CRP	c-reactive protein		
PsA	Psoriatic arthritis		
PK	Pharmacokinetic		
PD	Pharmacodynamic		
pSTAT3	Phosphorylated Signal transducer and activator of transcription 3		
nM	Nanmolar		
ePPND	Enhanced pre- and post-natal development		

Table of Contents

Section	Description	Page
1	Background & Rationale	4
Figure 1A & B	Outcomes of patients with cABMR and TG	5
1.1	IL-6/IL-6R Inhibition: An Important New Therapeutic Option for Prevention & Treatment of ABMR	5
Figure 2	Circulating Plasmablast Produce Copious Amounts of IL-6 which Activates Tfh differentiation	7
Figure 3	Tocilizumab Treatment Induces T_{reg} cells in RA patients	7
1.2	Experience with Anti-IL6R in a Mouse Model of Allosensitization	7
Figure 4A & 4B	Mousenized Anti-IL-6R Antibody in a Mouse Model of Allo-sensitization	8
Figure 5	Anti-IL6R Treatment Reduces Tfh formation and Induces T-regs in Mouse Model of Allosensitization	9
Figure 6A & 6B	Flow Cytometry Analysis of Blood from Nonhuman Primates Before and After Treatment with Tocilizumab	9
1.3	Therapeutic Use of Tocilizumab for Prevention of Antibody- Mediated Injury in Humans	10
Figure 7A & 7B	Our Tocilizumab Trial Design and Immunodominant DSA results	11
1.3.1	The Problem: Antibody Mediate Rejection	11
Figure 8	Pathogenic Mechanisms for ABMR in Renal Allografts	12
1.4	Is There a Role for IL-6 in Mediation of ABMR	12
Figure 9	Serum Cytokine Levels in Kidney Transplant Recipients with Normal Graft Function and Allograft Rejection	13
Figure 10A & 10B	IL-6 Expression in Kidney Tissue & IL-6+ Cells Increase in Allografts with ABMR	14
1.4.1	Impact of DSAs on Long-Term Allograft Survival	14
Figure 11A & 11B	Impact of DSAs on Long Term Allograft Survival and Mechanisms of Allograft Injury	14
Figure 12A & 12B,	Cedars-Sinai Long-term Outcomes	16
Table 1	Table 1: Characteristics predisposing to ABMR	17
1.4.2	Preliminary Data on Use of Tocilizumab for Treatment of ABMR in Humans	18
Figure 13A-E	Outcomes of Tocilizumab Patients Treated for cABMR and TG	19
2	Clazakizumab (anti-IL6) as a Potential Agent to Treat cABMR and TG	19
3	Primary Objectives	22
3.1	Major Secondary Objectives	22
3.2	Inclusion Criteria	22
3.3	Exclusion Criteria	22
4	Study Design & Methods	23
Figure 14	Clazakizumab Dosing Protocol for cABMR + TG	25
4.1	Study Analysis	25
4.2	Defining ABMR	25
4.3	Treatment of Allograft Rejection Episodes During the Study	25
4.4	Monitoring for AE/SAEs Post-Transplant in HS Patients	26
Figure 15A & 15 B	Proportion of Kidney Transplant Recipients who Developed Infections Post-Transplant	26
Figure 16	Viral PCR Positivity for CMV, EBV, and PolyomaBK Virus in Desensitized vs Standard Kidney Recipients	27
4.5	Safety Reporting of Adverse Events	27
4.5.1	Reporting of Serious Adverse Events Associated with Clazakizumab	28
4.6	Infection Prophylaxis Protocols and Viral Monitoring Post-Transplant	31
5	Dosing of Clazakizumab	31
5.1	Storage and Handling	32
5.1.1	Clazakizumab Overdosage	32
5.2	Dose Modification/Toxicity Management	32
5.3	Adverse Drug Reactions	32
Table 2	Neutropenia Risk Mitigation	30
Table 3	Thrombocytopenia Risk Mitigation	34
Table 4	Elevated Liver Enzymes and Hepatic Events	34
6	Therapy Stopping Points	37
7	Statistical Analysis	37
8	References	39
Appendix A	ABMR Visit Schedule	42
Appendix B	Send out Labs	43
Appendix C	DSMB Charter	43
Appendix D	Safety Reporting Forms	43

1.0 Background & Rationale

Antibody-mediated rejection (ABMR) is a unique, significant and often severe form of allograft rejection. ABMR is characterized by several pathologic variants that are often resistant to treatment with standard immunosuppressive medications. Significant advances have occurred in our ability to predict patients at risk for and to diagnose ABMR [1,2]. The pathophysiology of ABMR suggests a prime role for antibodies, B-cells and plasma cells. As a result, IVIG, rituximab, and/or plasmapheresis (PLEX) have been leveraged for the treatment of acute ABMR[3-7]. Despite the success of these therapies, post-transplant ABMR, chronic active ABMR (cABMR), and transplant glomerulopathy (TG) remain significant problems that are often unresponsive to current therapies[4]. Data from the Deterioration in Kidney Allograft Function (DeKAF) study show that most graft losses in the current era of immunosuppression have evidence of cABMR with positive C4d staining[2,8]. It is estimated that 5,000 renal allografts are lost each year in the US, primarily from cABMR and TG[9]. The current treatment paradigms rely on reduction of antibody levels to prevent ABMR. This raises the importance of maintaining immunosuppression and investigating novel methods to prevent and treat ABMR/cABMR that directly address the reduction of donor specific antibodies (DSAs) and antibody producing cells.

ABMR is frequently seen in patients receiving inadequate immunosuppression or who are noncompliant with anti-rejection medications and those who receive human leukocyte antigen (HLA)-incompatible transplants. In addition, TG is a known consequence of persistent DSA positivity which rapidly dissipates allograft function, resulting in graft failure and return to dialysis with attendant emotional consequences for the patients and financial consequences for the health care system[10-14]. No current therapy is FDA approved and patients are often treated with combination therapies that make analysis of efficacy difficult. Thus, there is a large unmet clinical need. To this end, it is imperative that novel therapies to prevent immunologic injury to the microcirculation be developed and studied in patients with cABMR and TG.

Interleukin-6 is a key cytokine which regulates inflammation and the development, maturation, and activation of T-cells, B-cells and plasma cells[15]. Excessive IL-6 production has been linked to a number of human diseases characterized by unregulated antibody production and autoimmunity[15,16]. We have shown IL-6/IL-6R interactions are critical for alloantibody generation in an animal model of alloimmunity[17]. Blockade of these interactions with an anti-IL-6R monoclonal results in significant reductions of alloantibodies, antibody production by splenic and bone marrow plasma cells, direct inhibition of plasma cell anti-HLA antibody production and induction of T_{reg} cells with inhibition of T-follicular (T_{fh}) cells. Thus, IL-6 shapes T-cell immunity and is a powerful stimulant for pathogenic IgG production [18,19].

This raises the importance of maintaining immunosuppression and investigating novel methods to prevent and treat antibody-mediated injury that directly address the reduction of DSAs and antibody producing cells.

Based on these observations, we feel there is a large unmet clinical need for new therapies to prevent and treat ABMR. This is also true for cABMR and TG that are now leading causes of chronic allograft failure. Despite our best efforts, we still anticipate an ABMR rate of approximately 25-40% in desensitized patients. From our experience, 80% of these episodes occur in the first 1-3 months post-transplant and can significantly impact long-term patient and graft survival. Current estimates of de novo DSA (dnDSA) induced ABMR suggest approximately 30% of kidney transplant patients are at risk for development of ABMR, cABMR and TG. Thus the scope of antibody-induced injury in the transplant population is significant and growing[9].

Recent data indicate that long-term outcomes of patients with cABMR and TG are very poor[20,21]. Redfield et al evaluated graft survival in 123 patients with cABMR. Once cABMR was diagnosed, 76 patients lost their allografts with a median graft survival of 1.9 years[20]. In addition, the graft survivals at 2 years for patients with cABMR without treatment was ~20%[20,21]. Data from this study is shown in Figure 1 A&B below:

Figure 1A Figure 1B

CABMR + TG Predicts Early Graft Failure

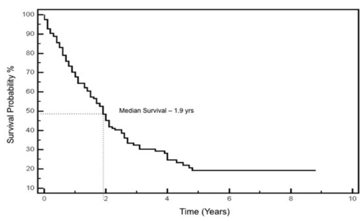

Robert R. Redfield, Thomas M. Ellis, Weixiong Zhong, Joseph R. Scalea, Tiffany J. Zens, Didier Mandelbrot, Brenda L. Muth, Sarah Panzer, Millie Samaniego, Dixon B. Kaufman, Brad C. Astor, Arjang Djamali
Current outcomes of chronic active antibody mediated rejection – A large single center retrospective review using the updated BANFF 2013 criteria
Human Immunology, Volume 77, Issue 4, 2016, 346–352
http://dx.doi.org/10.1016/j.humimm.2016.01.018

Current Therapies Show Some Beneficial Effects

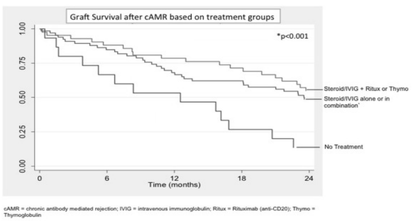

Robert R. Redfield, Thomas M. Ellis, Weixiong Zhong, Joseph R. Scalea, Tiffany J. Zens, Didier Mandelbrot, Brenda L. Muth, Sarah Panzer, Millie Samaniego, Dixon B. Kaufman, Brad C. Astor, Arjang Djamali
Current outcomes of chronic active antibody mediated rejection – A large single center retrospective review using the updated BANFF 2013 criteria
Human Immunology, Volume 77, Issue 4, 2016, 346–352
http://dx.doi.org/10.1016/j.humimm.2016.01.018

Figure 1A&B: Outcomes of patients with cABMR and TG are shown. Figure 1A shows outcomes for all 123 patients. By 1.9 years post-cABMR diagnosis, ~50% of grafts were lost. Figure 1B shows that current therapies using IVIG + steroids +/- rituximab are associated with improved outcomes at 2 years (~50% survival) compared to 20% with no treatment. These data indicate the poor prognosis seen with cABMR +TG that currently has no good therapies.

1.1 IL-6/IL-6R Inhibition: An Important New Therapeutic Option for Prevention & Treatment of ABMR

Interleukin-6 is an important mediator of inflammation and the development, maturation, and activation of T-cells, B-cells and plasma cells[22,23]. Excessive IL-6 production has been linked to a number of human diseases characterized by excessive and unregulated antibody production and autoimmunity[15,16]. Data are accumulating on the importance of inhibiting IL-6/IL-6R interactions in treatment of autoimmune disease and transplantation using the anti-IL-6R monoclonal tocilizumab.

Tocilizumab (Actemra®, Roche/Genentech, South San Francisco, CA, USA) is the first in class humanized monoclonal aimed at the IL-6 receptor (IL-6R). Tocilizumab binds to both soluble and membrane bound forms of the IL-6R receptor. Tocilizumab was recently approved by the FDA for treatment of rheumatoid arthritis and juvenile idiopathic arthritis. Since the introduction of the drug, other reports indicate that inhibition of the IL-6/IL-6R pathway may have significant benefits in SLE and other vasculitic disorders and reduces antibody producing cells in treated patients. Recent reports demonstrated a significant reduction in anti-ds-DNA antibodies and circulating plasma cells in SLE patients treated with tocilizumab[24,25]. Tocilizumab treatment can also result in a reduction of peripheral pre- and post-switch memory B cells in rheumatoid arthritis patients. These patients also showed significant reductions in IgG+ and IgA+ B cells and reduction in serum levels of IgG and IgA. Thus, B-cell hyper-reactivity is significantly impacted in humans treated with tocilizumab [24].

Reports have shown that tocilizumab also reduces antibody-producing cells, diminishes inflammatory markers, and improves clinical symptomatology in a number of other autoimmune diseases[15,16,26,27].Recent clinical observations and animal models have shown that IL-6 may be important in mediation of allograft rejection. IL-6 production increases in mouse allografts undergoing rejection and is responsible for allogeneic T-cell infiltration[22]. In addition, IL-6 deficiency, in combination with costimulatory pathway blockage by CTLA4-Ig induces graft acceptance[28]. It is accepted that IL-6 drives CD4 T-cells toward T-helper type 17 (Th17) phenotype while negating regulatory T-cell differentiation[15,24]. Neutralization of IL-6 reduces allograft rejection by allowing emergence of regulatory T cells (T_{reg})[22]. Recent evidence also indicates that IL-6 triggers IL-21 production by follicular T-helper cells (T_{fh}) driving B cell maturation to plasma cells during antibody responses[29,30]. Anti- IL-6R antibodies have shown significant reduction in graft-versus-host disease and allograft rejection in animal models associated with increased T_{reg} differentiation[31,32]. We have previously reported on the utility of a mousenized rat- anti-mouse IL-6 receptor monoclonal antibody (clone mMR16-1) in a mouse model of HLA.A2

sensitization[22]. The study found that anti- IL-6R treatment attenuates de novo DSA, including both immunoglobulin (Ig)M and IgG responses. We designed experiments to investigate the ability of anti-IL-6R to modify antibody recall responses to alloantigen which mimics the most relevant clinical scenario of highly sensitized patients. We hypothesize that anti- IL-6R suppresses donor-specific IgG response by blocking IL-6 signaling in plasma cells, which is critical for terminal differentiation of memory B cells to plasmablasts and for survival of long-lived plasma cells in the bone marrow (BM)[29]. Recent data[29] also suggests that IL-6 production by antigen-activated plasmablast is responsible for T_{fh} cell development and germinal center formation. This data was obtained in humans. Indeed, when patients with rheumatoid arthritis were examined, circulating T_{fh} and plasmablast numbers were elevated in patients with active disease. Treatment with tocilizumab significantly reduced T_{fh} cell numbers, IL-21 production by T_{fh} and circulating plasmablast (see Figure 2). Other recent data also show that tocilizumab induces significant increases in T_{reg} cells in patients with rheumatoid arthritis. This also correlated with responses to therapy[30]. These findings have potentially important implications in prevention and treatment of allograft rejection in humans.

Currently, there are two trials of tocilizumab for GVHD in humans. In addition, we have completed a trial of tocilizumab as a desensitization agent for HS patients awaiting renal transplantation and who had failed desensitization with IVIg + rituximab. This study is a Phase I/II study (NCT01594424) that is open label. Data on this trial were recently reported[33]. In this trial, we found that tocilizumab improved renal transplant rates in patients who had failed desensitization with IVIG + rituximab +/- plasma exchange (PLEX). Donor specific antibodies (DSAs) that were resistant to other therapies were significantly reduced with therapy and none of the transplanted patients showed antibody mediated rejection post transplant.

Figure 2: Circulating Plasmablast Produce Copious Amounts of IL-6 which Activates T_{fh} Differentiation

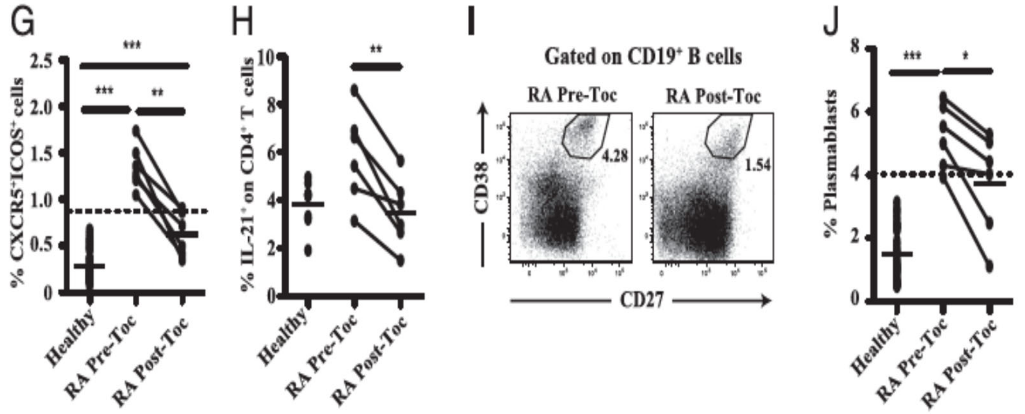

Figure 2: The figure shows that healthy individuals have low levels of circulating T_{fh} cells, but RA patients express higher levels with disease. Treatment with tocilizumab results in a dramatic reduction in T_{fh} cells, IL-21 production by T_{fh} cells and circulating plasmablast. The authors conclude that inhibition of IL-6/Il-6R interactions will likely reduce T_{fh} formation and reduce germinal center formation with subsequent reductions in pathogenic antibody production. The authors also demonstrated that circulating plasmablast produced copious amounts of IL-6 and was likely responsible for additional T_{fh} cell activation and germinal center formation. Treatment with tocilizumab resulted in a dramatic reduction in circulating plasmablast and IL-6 production[29].

Figure 3: Tocilizumab treatment induces T_{reg} cells in RA patients and is associated with remission of active disease. Note, T_{reg} cells arise after several months of therapy.

Anti-IL-6R Therapy Increases Treg Populations and is Associated With Remission in Rheumatoid Arthritis Patients

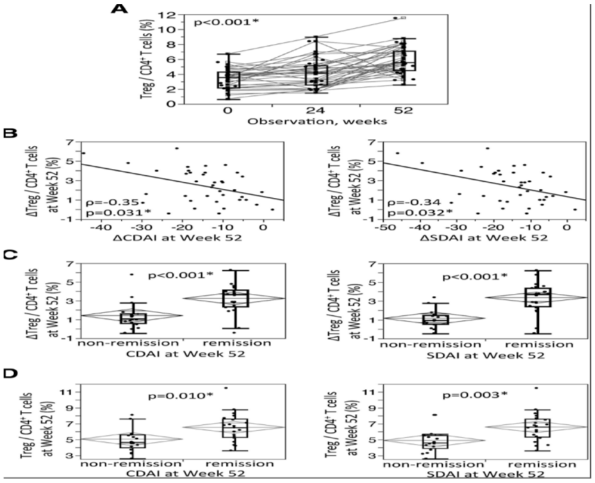

1.2 Experience with Anti-IL-6R in a Mouse Model of Allo-Sensitization.

Recent data from our group[22] investigated the ability of anti-IL-6R to modify immune responses to HLA-2 transgenic skin grafts. We investigated the ability to modify circulating anti-HLA-A2 antibody levels (DSA), plasma cell responses in the bone marrow and spleen of sensitized animals and the ability to modify T-cell subsets. The data are shown below:

Figure 4A & 4B: This figure shows data from our group using a mousenized anit-IL-6R antibody in a mouse model of allo-sensitization[22]. Briefly, after sensitization, we can detect anti-HLA-A2 antibodies in sera of animals. Anti-IL-6R significantly reduces circulating levels of anti-HLA-A2 antibodies. In addition, as shown here, there is a significant reduction of antibody producing plasma cells isolated from the bone marrow and spleens of sensitized animals (4A). More importantly, plasma cells producing anti-HLA-A2 antibody in the bone marrow are inhibited by anti-IL-6 therapy (4B). Addition of anti-IL-6 treatment of conditioned media of bone marrow plasma cells from untreated sensitized animals also significantly reduced anti-HLA-A2 antibody production.

Subsequent studies suggest that anti-IL-6R treatment is more effective than a mouse anti-mouse anti-CD20 in reducing rebound anti-HLA-A2 antibodies after re-exposure to HLA-A2 + skin grafts 90 days after 1st exposure. This simulates the sensitization situation we seen in humans who have highly allo-reactive T & B-cells at the time they present for desensitization.

Figure 4A: Anti-IL6R Supresses Plasma Cells Ig Production in HLA Allosensitized Mouse Model

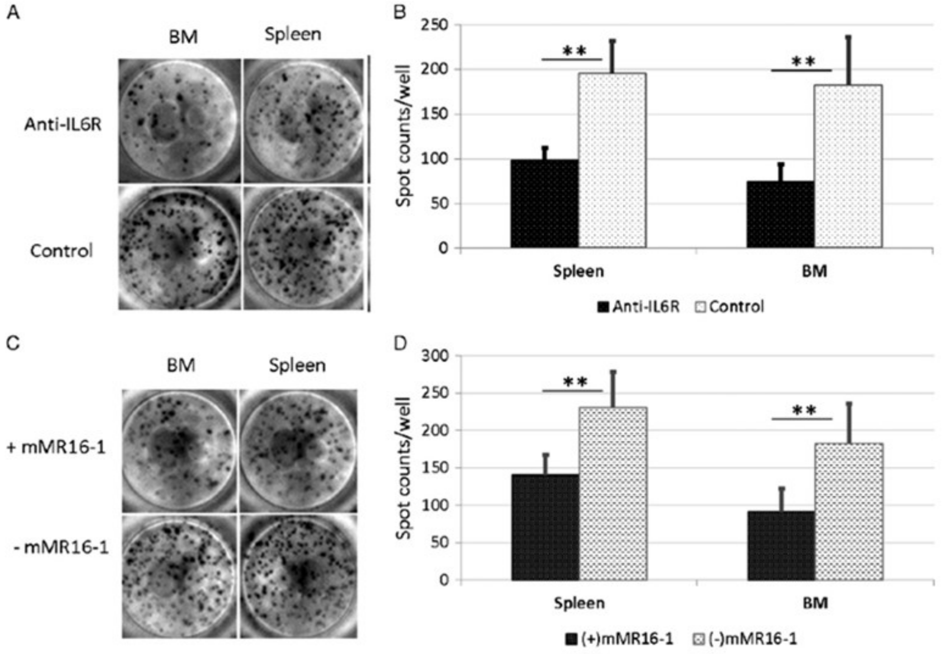

Figure 4B: M-MR16-1 (Anti-IL-6R) Significantly Reduces Anti-HLA Antibody Production by Plasma cells Isolated from Bone Marrow of Sensitized Mice

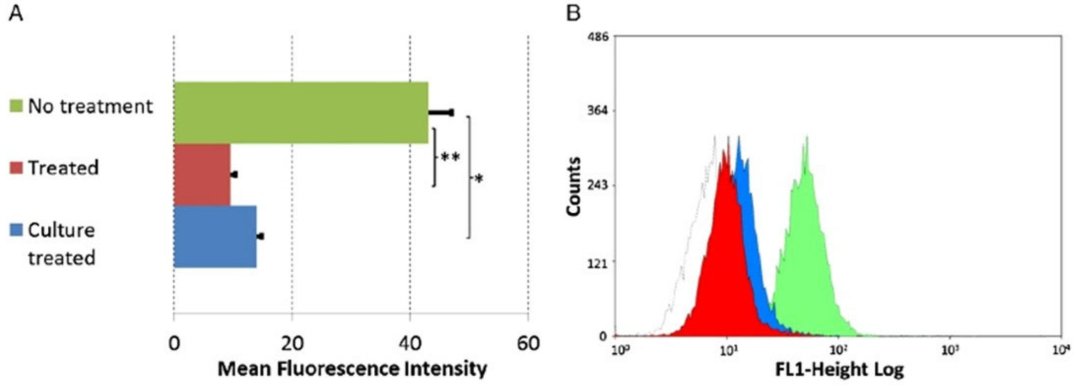

Figure 5: This figure shows that in addition to the ability of anti-IL-6R treatment to reduce plasma cell IgG and anti-HLA-A2 production, there are significant alterations in the cellular compartments as well. First, anti-IL-6R treatment of sensitized animals is associated with a deviation of T-cell subsets to a T_{reg} profile. This is associated with a significant reduction in the T_{fh} markers (IL-21 and CXCR5). This data suggests that anti-IL-6R therapy modifies T-cell responses to allo-antigens resulting in a diminution of inflammatory T-cell responses and likely reduction in T_{fh} induced germinal center formation[22].

Figure 5

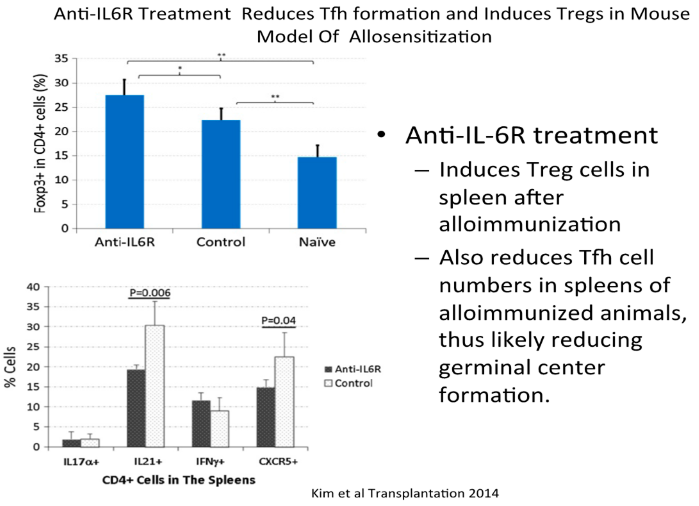

In addition, recent data from a non-human primate (NHP) model of lung transplantation suggests that tocilizumab is critical for long-term rejection-free survival. Tocilizumab treatment was associated with an increase in T_{reg} cells and inhibition of DSA generation post-transplant[34]. Of interest in this model is the importance of T-cell depletion at transplant to maximize the induction of T_{regs} by tocilizumab. This is also similar to the protocol we used post-transplant in our highly-HLA sensitized patients receiving desensitization with IVIG + tocilizumab where we saw rapid reductions of DSAs and no evidence of ABMR on protocol biopsies performed at 6 months[35]. Data are shown in Figure 6A & 6B below.

Figure 6A Tocilizumab Induces Treg Cells Post-Transplant in Primate Model of Lung Transplantation

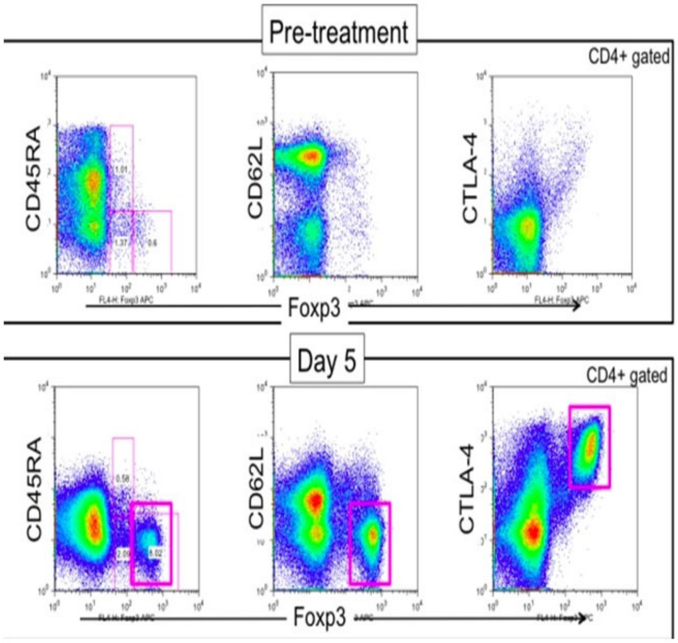

Figure 6B Tocilizumab + ATG Induction Dramatically Improves Lung Allograft Survival in Incompatible Primates

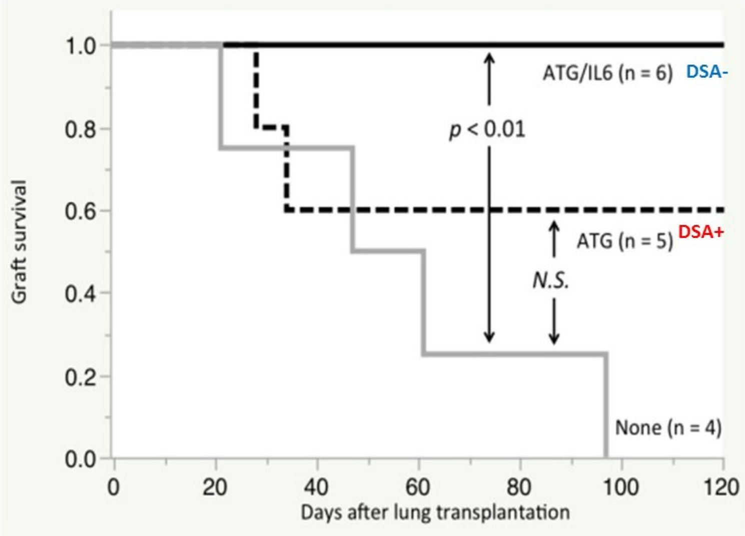

Figure 6A & 6B: Figure 6A shows the flow cytometry analysis of blood from nonhuman primates before and after treatment with tocilizumab. Briefly, tocilizumab treatment results in a dramatic increase in T_{reg} cells in the circulation of the NHP animals. Figure 6B shows the results of lung transplantation in NHP who received standard treatment with tacrolimus + steroids + MMF (SOC), ATG + SOC and ATG + tocilizumab + SOC. Briefly, animals treated with SOC had all lost their grafts by 100 days with 20% survival at 60 days. All animals were also DSA+. Those receiving ATG + SOC had 60% survival at 120 days and were also DSA+. However, those that received ATG + tocilizumab + SOC had 100% survival at 120+ days and were DSA-. We also know from this unpublished data that the animals receiving both ATG + tocilizumab had significantly greater increases in T_{reg} cells compared to use of either agent alone. Thus the increase in T_{reg} is likely responsible for the improved graft survival and lack of DSA development post-transplant.

1.3 Therapeutic Use of Tocilizumab for Prevention of Antibody- Mediated Injury in Humans

As mentioned above, alloantibodies are a major deterrent to access to and success of life-saving organ transplants. Despite advancements in desensitization, designing efficient and effective means of removal of pathogenic HLA antibodies remains a significant medical challenge. Preliminary data suggests that tocilizumab exhibits a broad range of immunomodulatory actions that could address the allo-antibody response to allografts that are so destructive.

We recently completed a trial assessing the ability of tocilizumab to modify alloantibody responses and improve transplant rates in highly-HLA sensitized patients with end-stage renal disease (ESRD) who had failed desensitization with IVIG + rituximab +/- PLEX[33]. Data from this study are shown below in Figure 7A & 7B.

This figure summarizes data from a recently completed open labeled Phase I/II clinical trial of tocilizumab in highly-HLA sensitized patients awaiting kidney transplants. All patients had had previous renal transplants and were resistant to desensitization with IVIG + rituximab +/- PLEX. Ten patients were entered and six transplanted during the study. Two of the non-transplanted patients were withdrawn from the study due to non-compliance with the protocol. Thus, six of eight patients remaining in the study received transplants. The figures above show the course of DSAs before therapy with tocilizumab, at transplant and 12 months later. As can be seen, significant reductions in immunodominant DSAs was seen in all transplanted patients. This was most significant at 12M post-transplant. It is important to note that all patients received an additional 6 monthly doses of tocilizumab after transplant. Protocol biopsies at 6M showed no evidence of rejection in any patient. The protocol design and results are shown below:

Figure 7A and 7B Tocilizumab Protocol for Desensitization Pre- & Post-Transplant

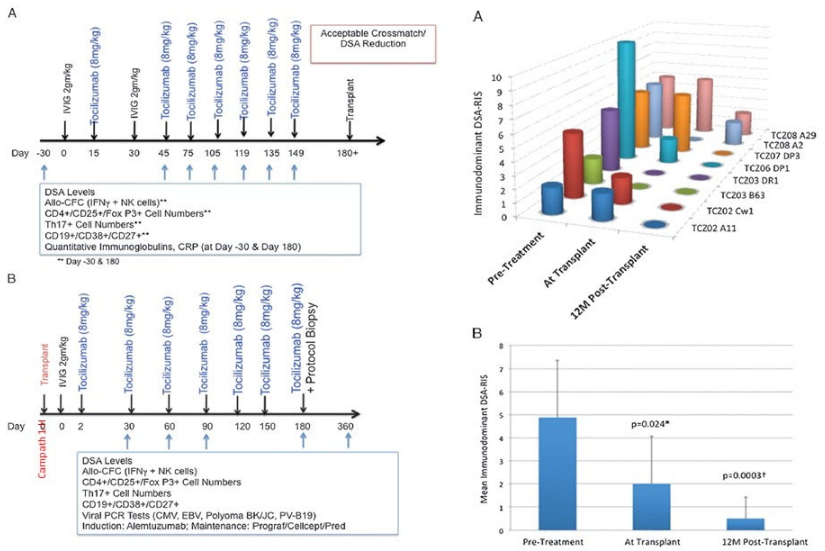

Figure 7A & 7B: Our original trial design for desensitization of highly-HLA sensitized ESRD patients using tocilizumab is shown. Briefly, patients entered into this study received desensitization with IVIG + tocilizumab for up to 6 months. If renal transplantation was accomplished, patients received an additional 6M of tocilizumab after initial induction with alemtuzumab 30 mg/SQ and IVIG post-transplant. DSAs and other immune parameters were measured. Importantly, DSAs present pre-transplant were reduced by tocilizumab + IVIG treatment. Post-transplant tocilizumab resulted in a marked reduction in DSAs that were analyzed at 6M and 12M post-transplant (Figure 7B). Biopsies performed at 6M post-transplant showed no patients had ABMR, TG or cABMR. These results are similar to that reported in the NHP models above since T-cell depletion + tocilizumab + SOC was used post-transplant.

1.3.1 The Problem: Antibody Mediated Rejection

DSAs (Anti-HLA antibodies) have a strong and universally deleterious impact on mediation of allograft injury and loss. As depicted in Figure 8 below, pre-formed or de novo DSAs activate complement, induce endothelial cell proliferation antigens and mediate ADCC resulting in a progression of allograft dysfunction and loss. More than 5000 renal allografts are lost each year in the U.S., approximately 75-80% to antibody mediated injury[9,36]. Thus, understanding the pathophysiology of ABMR and B-cell activation are critical to improving the longevity of existing allografts and development of successful strategies for desensitization to prevent ABMR. With this understanding, new drugs can be developed to address relevant pathways. Currently, there are no approved drugs for prevention or treatment of ABMR. This represents one of most pressing unmet needs in transplant medicine.

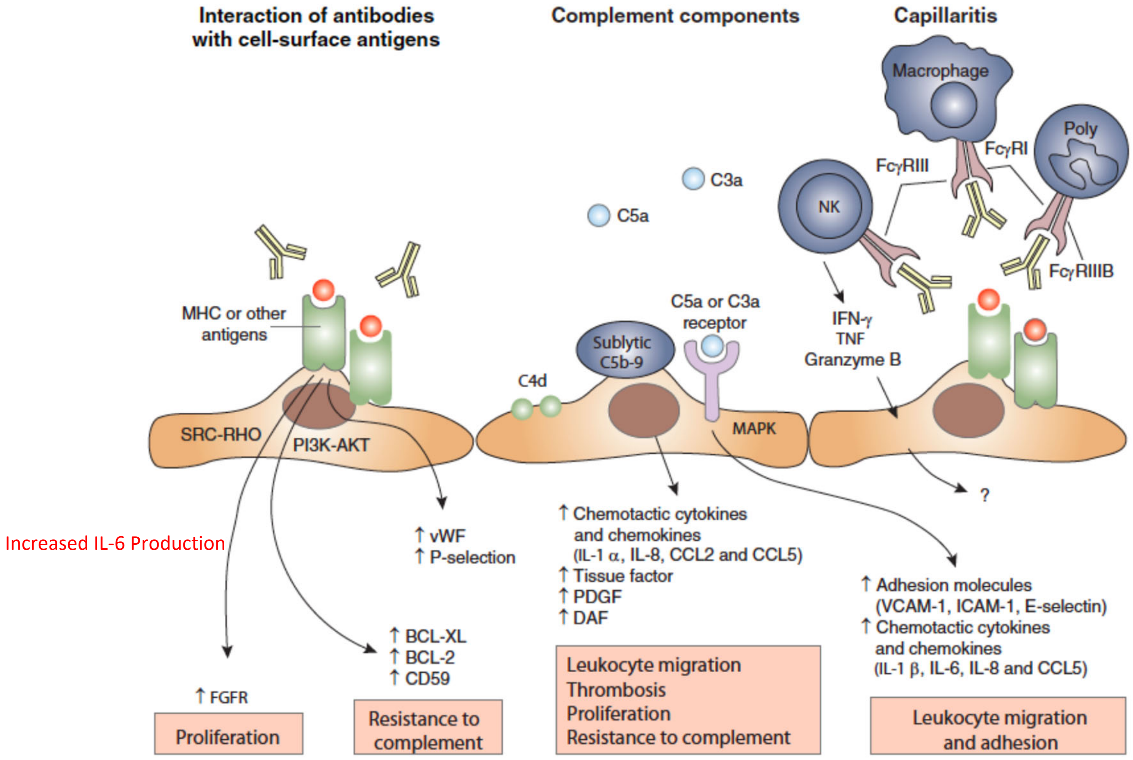

Figure 8. This figure shows the relevant pathogenic mechanisms responsible for ABMR in renal allografts. First and most dominant is CDC where DSA-mediated complement activation initiates an inflammatory cascade that results in severe injury to the allograft endothelium. ADCC mediated by NK cells, macrophages, and PMNs also causes severe injury to the allograft[1]. In addition, DSAs can activate endothelial cell IL-6 transcription without ADCC or CDC through direct interactions with HLA molecules on endothelial cells.

1.4 Is There a Role for IL-6 in Mediation of ABMR

We have performed extensive investigations into the role of IL-6 overexpression in the mediation of ABMR. Our first studies have centered on measurement of serum cytokine levels in peripheral blood of ESRD patients awaiting kidney transplant. This data is not yet published but is shown below.

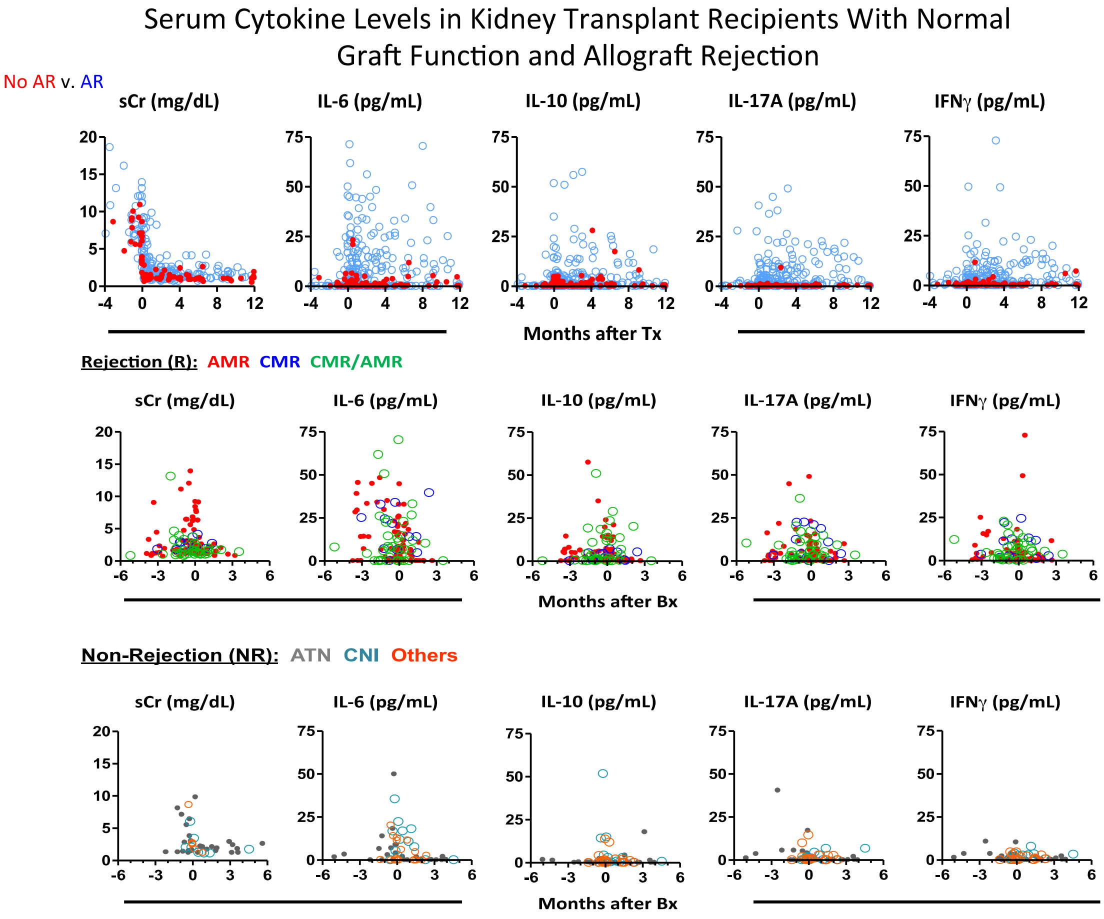

Figure 9: This figure shows the relationship of serum cytokines measured at various times post-transplant. Briefly, in panel A, serum cytokines are shown for patients who had for cause biopsies (blue) vs. those who did not have biopsies. As can be seen, the IL-6 levels are quite low in patients with quiescent allografts. However in Panel B, patients with ABMR show significant elevations of IL-6 serum levels in concert with ABMR onset. The X axis shows time before, at and after biopsies. IL-6 levels appear to diminish with treatment of ABMR. Panel C shows cytokine levels in patients who had biopsies that did not show allograft rejection. These data suggest that elevations of serum IL-6 levels could be used as an early marker for allograft dysfunction mediated by antibody injury.

We next endeavored to determine if IL-6 is expressed in the biopsies of patients undergoing allograft rejection. To accomplish this, we examined renal biopsy material from patients with normal kidneys, patients with cellular rejection and patients with ABMR. Sections were stained with anti-sera directed at IL-6 and evaluated by morphometric scanning microscopy. The data are shown below. Briefly, our investigations showed that the number of IL-6+ cells were significantly increased in biopsies demonstrating ABMR. This suggests a possible role for IL-6/IL-6R interactions in mediating ABMR and enhanced DSA production. Along with the data presented above which demonstrates elevated levels of IL-6 in the sera of patients with ABMR these findings suggest the possibility of IL-6 blockade as a potentially important therapy in management of ABMR and possibly cABMR and TG.

Figure 10A. Figure 10B.

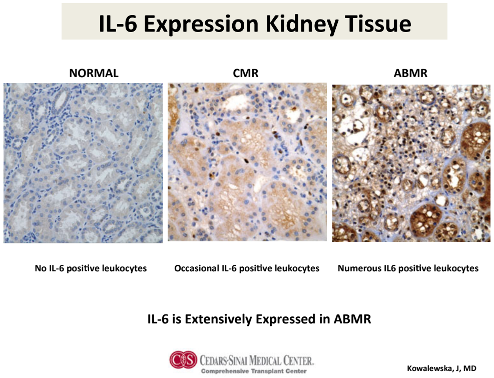

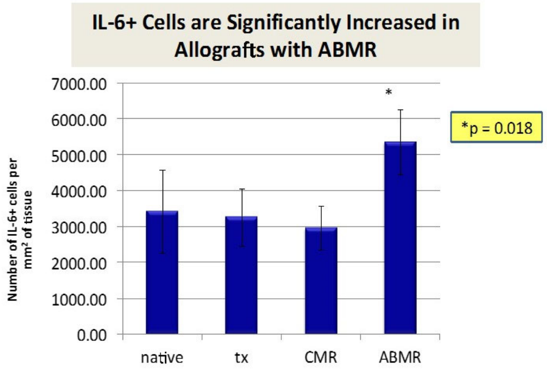

Morphometric analysis of IL-6 staining in native kidneys (native, n= 6 with thin basement membrane disease), transplants without rejection (tx, n=9), transplants with cell mediated rejection (CMR, n=12) and antibody-mediated rejection (ABMR, n= 11)

Figure 10A&10B: Figure 10A shows representative staining of normal kidney tissue, tissue from a patient with cellular rejection and a biopsy from a patient with antibody-mediated rejection. In this instance, there are numerous IL-6+ cells in the biopsy of ABMR compared with CMR and normal tissue. Figure 10B shows data from a larger analysis of ABMR biopsies compared to other diagnoses. Using morphometric scanning analysis, we were able to show a significant increase in IL-6 expression in biopsies with ABMR. These data suggest that IL-6 might play an important role in antibody-mediated injury to allografts.

1.4.1 Impact of DSAs on Long-Term Allograft Survival

Data shown in the composite graphs below show the impact of DSAs, especially C1q+ or complement activating DSAs on long-term graft survival. This important paper from the French group codified the importance of C1q+ DSAs in mediating the more severe forms of ABMR and cABMR. Another important factor shown in figure 11B is the impact of DSAs on accelerating atherosclerotic changes in the allograft which quickens demise. This may also be related to IL-6 induction in endothelial cells by DSAs that results in obliterative vasculopathy as was previously reported[37]. Thus, antibody-targeted therapies would be of importance in minimizing these manifestations of DSA injury.

Figure 11A

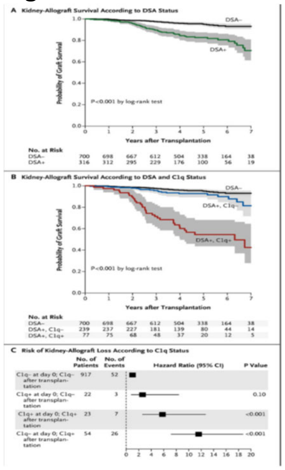

Figure 11B

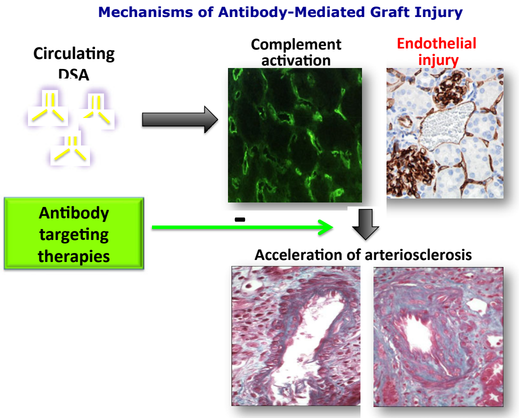

Figure 11A & 11B: Figure 11A shows the impact of DSAs on long term allograft survival in patients transplanted in France[38]. These investigators showed that DSAs have a negative impact on long-term graft survival, but when segregated by ability to activate complement (C1q+ DSAs), the impact was significantly magnified. In fact, those who developed C1q+ DSAs post-transplant has a 12fold greater risk for allograft loss than C1q- DSA patients. Figure 11B shows the

mechanisms of alloantibody injury to grafts which include complement activation endothelial injury through direct actions of alloantibody on ENDATs and ADCC as well as induction of accelerated atherosclerosis in allografts[9].

Clearly, the most important way to approach antibody mediated allograft injury is through the development of antibody-targeted therapies. This would address all pathogenic mechanism associated with alloantibody and allow grafts to continue functioning for much longer periods of time. This would improve the length and quality of life of allograft recipients and reduce costs to the health care system.

Figure 12A & 12B below show data from our center on the long-term outcomes in patients who experienced ABMR after desensitization v. those who did not.

Figure 12A: Figure 12B:

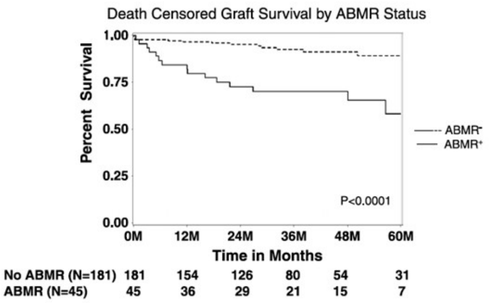

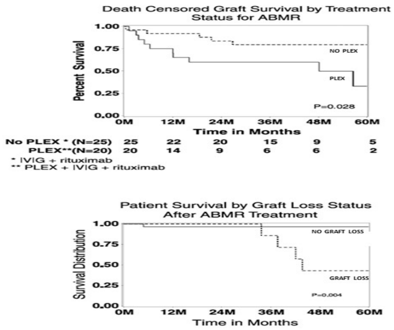

Figure 12A & 12B. We retrospectively reviewed the outcomes of 226 patients who underwent desensitization with IVIG + Rituximab +/- PLEX at Cedars-Sinai Medical Center. Patients were divided into two groups. Group 1 had no ABMR (80%) while Group 2 experienced ABMR (20%). As can be seen in Figure 12A, the long-term outcomes in HS patients who are ABMR+ are significantly worse than those who were ABMR-. In fact, data for the 5 year outcomes of Group 1 are similar to non-sensitized patients in the UNOS data base. This suggest prevention of ABMR is critical for an excellent long-term outcome of allografts. Assessment of factors associated with risk for ABMR showed that re-transplantation, female gender, ESRD caused by glomerulonephritis and presence of stronger class I and class II DSAs at time of transplant were most important. (Table 1). Figure 12B shows the difficulty in treating ABMR after transplant in HS Patients. Patients were treated with IVIG + rituximab or IVIG + rituximab + PLEX. Those treated with IVIG + rituximab did better, but this likely reflects a selection bias as more severe cases were treated with PLEX. Clearly, the most important thing we can accomplish is to develop new strategies to prevent ABMR altogether to allow the long term benefits of transplantation to be manifest in these patients. In addition, this data points out that current treatments for severe, early onset ABMR with rapid reduction in renal function, represents an unmet medical need as long-term outcomes for graft survival are poor and, patient survival after graft failure from ABMR is significantly worse than those who responded to treatment[39].

Characteristics Predisposing to ABMR after Desensitization

Characteristic	No ABMR (n=181)	ABMR (n=45)	P
Age, years	49.2±13.0	41.0±11.4	0.0001
Male sex	54 (29.8)	22 (48.9)	0.015
Deceased donor	121 (66.9)	32 (71.1)	0.584
Race			
Caucasian	78 (43.1)	20 (44.4)	0.870
African American	38 (21.0)	15 (33.3)	0.080
Hispanic	45 (24.9)	6 (13.3)	0.097
Asian	15 (8.3)	2 (4.4)	0.381
Other	5 (2.8)	2 (4.4)	0.559
Primary disease			
Hypertension/diabetes mellitus	84 (30.3)	20 (44.4)	0.812
Glomerulonephritis/vasculitis	53 (27.2)	24 (53.2)	0.002
Congenital cystic/dysplastic	10 (5.5)	2 (4.4)	0.772
Others	43 (23.8)	13 (28.9)	0.475
Unknown	23 (12.7)	3 (6.7)	0.255
Previous transplants			
0	90 (50.0)	9 (20.5)	0.0003
1	68 (37.8)	20 (45.5)	0.397
≥2	22 (12.2)	15 (34.1)	0.0005
Other sensitizing events			
Pregnancy	80 (47.6)	12 (27.9)	0.032
Blood transfusions	92 (54.8)	19 (44.2)	0.301
Time on waitlist (mo)	92.9±49.6	131.3±66.3	0.0004
Time to transplant (mo)	4.0±6.9	3.0±3.7	0.449
PRA % at transplant			
Class I	70.3±44.3	83.11±23.92	0.151
Class II	60.9±34.3	59.5±40.6	0.903
Class I >80%	109 (61.6)	32 (71.1)	0.236
Class II >80%	68 (41.2)	24 (57.1)	0.064
T-cell FCMX (MCS) at transplant	60.2±71.3	130.4±103.9	<0.001
Pronase	31.5±76.1	71.2±90.0	0.019
B-cell FCMX (MCS) at transplant	212.8±143.1	249.4±135.8	0.136
Pronase	145.6±123.6	216.0±109.8	0.007
Positive DSA at transplant, any			
Class I only	44 (24.3)	7 (15.6)	0.208
Class II only	26 (14.4)	5 (11.1)	0.570
Both Class I & Class II	43 (23.7)	28 (62.2)	<0.001
None	68 (37.6)	5 (11.1)	0.0007
HLA Matches			
Zero match	36 (19.9)	14 (31.1)	0.105
1-2 Ag Matches	104 (57.5)	24 (53.3)	0.617
≥3 Ag Matches	41 (22.7)	7 (15.6)	0.297
Induction			
Alemtuzumab	143 (79.0)	41 (91.1)	0.061
Daclizumab	28 (15.5)	3 (6.7)	0.124
Thymoglobulin	6 (3.3)	1 (2.2)	0.704
Simulect/basiliximab	4 (2.2)	0 (0.0)	0.314

1.4.2 Preliminary Data on Use of Tocilizumab for Treatment of ABMR in Humans

Based on the preliminary investigations in animal models and humans, we began treating patients with ABMR who had failed treatment of IVIG + rituximab +/- PLEX. In April 2011, we treated our 1st patient who had onset of severe ABMR post-transplant that was unresponsive to treatment with PLEX + IVIG (SOC). The patient received tocilizumab 8mg/kg initially, then monthly X 4 more doses and had a complete recovery with SCr 1.3mg/dl now nearly 6 years on. Since this initial patient, we have treated 36 patients with chronic, active ABM (cABMR) and TG. These patients have a very bad prognosis, with ~ 50% graft loss by 2 years post-biopsy. In 2011, we began treatment of patients with cABMR and TG using monthly tocilizumab at 8mg/kg. Treatment was carried out for 6-24 months. Patients were monitored for DSAs, graft/patient survival and maintenance of renal function. Outcomes were compared to 39 patients with similar biopsy findings who were treated with standard-of-care (IVIg + rituximab + PLEX) Data for these patients is show in Figure 13 below. Briefly, treatment with tocilizumab was associated with a significant improvement in patient and graft survival of the ensuing 6 years. Patients in the cABMR + TG group had a graft survival at 6 years of 82% v. 20% for the SOC group (Figure 13C). In addition to the improved outcomes, patients treated with tocilizumab also showed stabilization of renal function, reduced DSAs and improvement in biopsy parameters of cABMR one year after initiatlon of tocilizumab therapy[40]. Data for these patients compared with SOC are shown in Fig 13A-E below. Briefly, outcomes for all tocilizumab patients, including graft survival at 6 years (13A), graft survival for those with TG (13B), graft survival for patients who lost their graft more than 3 months post-biopsy and patient survival all show significant improvement in those treated with tocilizumab compared to SOC. Figure 13E shows that biopsies performed in 9 patients 1 year after initiation of tocilizumab therapy showed significant improvement in C4D+ scores and g + ptc scores.

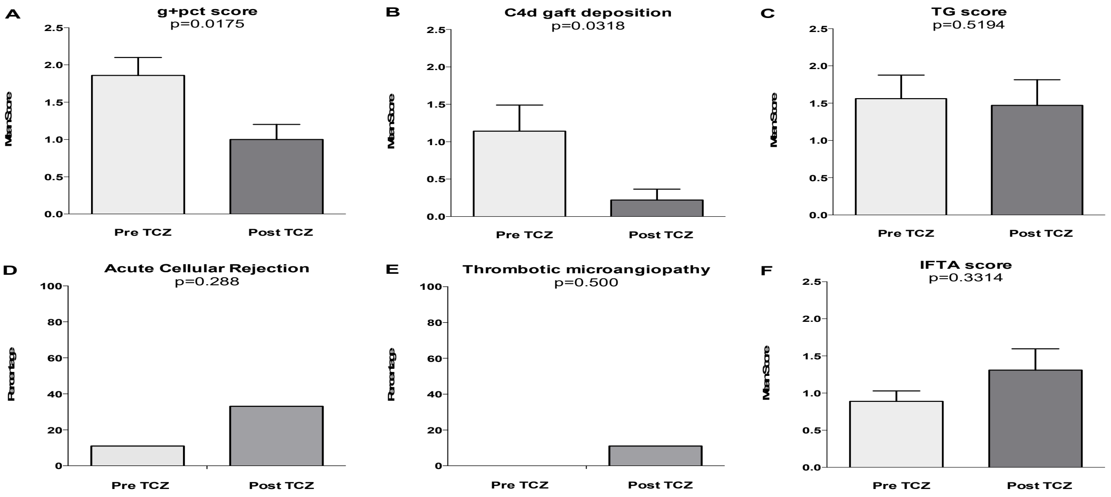

Figure 13A-E: This figure shows the outcomes of patients treated with tocilizumab for cABMR and TG are shown and described above. 36 patients were treated with tocilizumab monthly for 6-25 months and 39 patients received standard of care (SOC) treatment (IVIG + rituximab +/- PLEX) and constitute the retrospective cohort. Tocilizumab patients have been followed for up to 6 years. There are now 54 patients in this cohort. This data supports the idea that interruption of the IL-6/IL-6R signaling pathway may be important in treating patients with severe cABMR and TG mediated by HLA antibodies. In addition, we see an important reduction of DSAs over time that would indicate a potential benefit in desensitization treatments. Figure 13A shows the graft survival of all patients from index biopsy. Tocilizumab patients have superior graft survival. In 13B, we see the survival of patients with TG where 82% of tocilizumab treated patients' graft were functioning at 6 years. Figure 13C shows the graft survival for patients who lost their grafts >3M after index biopsy and figure 13D shows a near significant benefit in increasing patient survival. In figure 13E, 9 patients who received tocilizumab at index biopsy and had repeat biopsies at 1 year are shown. We compared scores for inflammation and chronicity. Briefly, tocilizumab patients showed reductions in C4d+ staining and g + ptc scores, features which are commonly associated with a worse prognosis.

2.0 Clazakizumab (anti-IL-6) as a Potential Agent to Treat cABMR and TG

Clazakizumab (CSL Behring LLC., King of Prussia, PA) is a humanized monoclonal antibody aimed at the cytokine IL-6 ligand. Clazakizumab has been evaluated extensively in patients with rheumatoid arthritis, but has not yet been approved by the FDA for any condition. Since the introduction of IL-6/IL-6R blocking drugs, reports indicate that inhibition of the IL-6/IL-6R pathway may have significant benefits in SLE and other vasculitic disorders and reduces antibody producing cells in treated patients. There is currently no information for Clazakizumab in HS patients awaiting incompatible (HLAi) transplants or for treatment of antibody-mediated rejection.

Clazakizumab is a genetically engineered humanized immunoglobulin G1 (IgG1) antibody that binds to human IL-6 with an affinity of 4 pM. Using multiple assays for signaling and cellular functions in response to IL-6 alone (to measure classical signaling) and a combination of IL-6 and sIL-6R (to measure trans-signaling), it was demonstrated that

clazakizumab is a potent and full antagonist of IL-6-induced signaling as measured by phosphorylation of signal transducer and activator of transcription 3 (STAT3), as well as cellular functions such as cell proliferation, differentiation, activation, B-cell production of immunoglobulins, and hepatocyte production of acute phase proteins (C-reactive protein [CRP] and fibrinogen). In addition, clazakizumab is shown to be a competitive antagonist of IL-6-induced cell proliferation. This in vitro pharmacological profile supports the potential of clazakizumab to impact multiple immune and non-immune cellular processes that are central to disease pathogenesis, and thus, to offer a new therapeutic modality in the treatment of autoimmune diseases and other IL-6 mediated diseases.

The clazakizumab development program includes a comprehensive nonclinical development program and clinical studies conducted in healthy subjects and in subjects with RA, psoriatic arthritis (PsA), Crohn's disease, graft-versus-host disease (GVHD), and oncology. To date, no studies with clazakizumab have been conducted in subjects with highly sensitized patients undergoing renal transplant, although supporting safety data are available from the previous clinical studies.

Nonclinical Studies
A comprehensive nonclinical development program has been completed. Clazakizumab was shown to be a potent inhibitor of IL-6-induced acute phase proteins. In pharmacokinetic (PK)/pharmacodynamic (PD) studies, a single dose of clazakizumab resulted in full inhibition of IL-6 activity as measured by the inhibition of IL-6-induced phosphorylated STAT3 (pSTAT3) activity in whole blood treated ex vivo with IL-6. The results of this functional PD assay correlated with drug exposures where full inhibition of pSTAT3 activity was observed when drug levels exceeded 50 ng/mL (approximately 0.3 nM). In a tissue cross-reactivity study, tissue binding of clazakizumab was observed in multiple tissues in both human and cynomolgus monkey, was generally cytoplasmic in nature, and was consistent with the known expression of IL-6 by cells and tissues. Results from both single- and repeat-dose nonclinical toxicology studies of up to 6 months in cynomolgus monkeys demonstrated an acceptable safety profile for clazakizumab. In a preliminary enhanced pre- and post-natal development (ePPND) study conducted in cynomolgus monkeys, an increase in the number of monkeys with retention of the placenta at parturition was observed at clazakizumab doses of 3 mg/kg (n=2) and 30 mg/kg (n=3), corresponding to doses 11 and 110 times the planned human dose of 50 mg. There were no other safety findings of clinical concern.

Clinical studies
Clinical studies have been conducted in healthy subjects and in the following patient populations: RA, PsA, CD, graft-versus-host disease (GVHD), and oncology. These clinical studies include a total of 1,223 subjects, of which 888 subjects were exposed to clazakizumab with doses ranging from 1 mg to 640 mg given by either intravenous (IV) or subcutaneous (SC) injection for up to 48 weeks.

<u>Clinical Pharmacology</u>

Following the administration of clazakizumab as a 1-hour IV infusion, the PK of clazakizumab were linear over the dose ranges of 30 mg to 640 mg in healthy subjects and 80 mg to 320 mg in subjects with RA as indicated by consistent clearance at these dose levels. The T-half of clazakizumab at all doses was very similar in healthy male subjects and in subjects with RA and was consistent with that expected for a humanized IgG1 antibody. Across the doses studied, the mean T-half of clazakizumab ranged from 19.5 to 31.0 days in healthy male subjects and from 26.4 to 30.9 days in subjects with RA. The T-half of clazakizumab after SC administration in healthy male subjects was similar to the IV administration. In a Phase 1 study comparing IV and SC dosing in healthy male subjects, the mean T-half of clazakizumab was 30.7 days after a single IV dose and, 31.1 to 33.6 days after SC administration. The bioavailability of clazakizumab after SC administration was 60% of the IV formulation. As expected, Cmax was lower and Tmax was longer for the SC administration relative to IV administration.

Population PK analysis of the data from clinical studies in RA, PsA and healthy subjects have indicated that body weight affects the PK of clazakizumab such that both clearance and central volume of distribution increase with increasing body weight. Therefore, heavier subjects will have lower drug exposure compared with less heavy subjects.

Clinical Efficacy and Safety Studies

Efficacy and safety data for clazakizumab is available from clinical studies conducted in RA, PsA, and oncology. Studies conducted in GVRD and Crohn's disease were prematurely terminated due to safety concerns and therefore no efficacy conclusions are available for these studies.

In Phase 2 studies in RA and PsA, doses from 5 mg SC once every 4 weeks (Q4W) up to 320 mg IV once every 8 weeks were significantly effective with clinical response evident as early as 12 weeks post treatment. One study in RA also demonstrated that the efficacy of clazakizumab is comparable or may be better than the standard of care treatment (adalimumab + methrotrexate (MTX)) in RA.

Efficacy with clazakizumab was not shown in the 2 Phase 2 studies in oncology (head and neck cancer and non-small cell lung cancer).

Two studies were terminated prematurely due to safety concerns. A Phase 2 study in Crohn's was terminated early because of GI perforation in 3 subjects who had received clazakizumab and this indication is no longer being studied. Although these subjects had multiple confounding medical issues, and the disease itself has an inherent risk of mucosal perforation, gastrointestinal perforations were also observed during the clinical studies with tocilizumab in patients with RA. Gastrointestinal perforation is a recognized risk of anti-IL-6 mAbs. After only 3 subjects were enrolled, a study in subjects with GVHD was also prematurely terminated due to 2 subjects experiencing similar serious adverse events (SAEs) (i.e., acute renal failure) which led to death. Both subjects had severe GVHD disease at the time of death.

Overall, the safety findings from the clinical studies conducted with clazakizumab to date are consistent with the known effects of blocking the IL-6 pathway (see Actemra® prescribing information]). Identified risks associated with clazakizumab administration include the following: infections, liver function test (LFT) abnormalities, changes in hematology parameters (i.e., neutropenia and thrombocytopenia), dyslipidemia (i.e., hypercholesterolemia and hypertriglyceridemia), and gastrointestinal perforations.

For further details regarding clinical studies with clazakizumab, please consult the Investigator's Brochure.

3.0 Primary Objectives

This study will be an open label design to assess the safety and efficacy of clazakizumab 25 mg SC given every 4 weeks (30 days) for a total of 6 doses in eliminating DSAs and stabilizing clinical features of cABMR in patients who have biopsy proven severe cABMR +TG. If improvements are seen in pathologic features of antibody mediated rejection from this biopsy compared to index biopsy, we will offer patients the opportunity to continue clazakizumab administrations monthly for an additional 6 months. A one year biopsy will then be performed at the end of the study. Safety determinations will be aimed at assessments of any side effects associated with clazakizumab administration and risk for infectious complications associated with clazakizumab therapy. Limited efficacy determinations will include assessment of composite surrogate end points including patient and graft survival, stabilization of the clinical features of cABMR (eGFR, SCr) after clazakizumab treatment, and safety throughout study duration.

Long term primary objectives include:

1. assess the safety and efficacy of clazakizumab 25 mg SC given every 4-8 weeks in eliminating DSAs and stabilizing clinical features of cABMR in patients who have biopsy proven severe cABMR +TG
2. assess composite surrogate end points including patient and graft survival, stabilization of the clinical features of cABMR (eGFR, SCr) after clazakizumab treatment, and safety throughout study duration

3.1 Major Secondary Objectives

In addition to our efforts to determine if clazakizumab treatment can significantly reduce or eliminate the severity of cABMR episodes and modify pathologic features (Banff 2015) at the end of 6 months of clazakizumab therapy, we will assess allograft function up to 6-12 M post-transplant, determine renal function using SCr, MDRD GFR (Schwartz equation will be used to estimate CrCl for patients under 18 years of age) calculations and DSA levels. A protocol biopsy will be performed at 6M post-clazakizumab therapy and 12M (for patients who receive 12 doses of clazakizumab). We will also record any late cABMR episodes after clazakizumab therapy. In addition, several immunologic determinations will be assessed at time points before and after initiation of clazakizumab therapy. These include the following:

- Assessment of T_{reg} cells (CD4+,CD25+,FoxP3+CD127^{dim})
- Assessment of T_{fh} cells (CD4+,ICOS+,CXCR5+, IL-21+)
- Assessment of circulating plasmablast (CD19+, CD38+,CD27+, IL-6+)
- Assessment of CRP, IL-6 levels and Quantitative immunoglobulins by ELISA pre- and post-treatment for cABMR
- Viral PCR monitoring by center protocol for EBV, CMV and Polyoma BK

This study protocol is shown in Figure 14. These secondary end points will help us understand the biology of alloimmune responses to allografts and to determine the ability and mechanisms of clazakizumab's beneficial effects. We will also monitor viral PCRs as per standard-of-care. Long term secondary objectives include:

1. determine renal function using SCr, eGFR
2. DSA levels
3. A protocol biopsy will be performed at 12M, 24M post-clazakizumab LTE.
4. record any late cABMR episodes after clazakizumab therapy.
5. Assessment of CRP and IgG
6. Viral PCR monitoring by center protocol for EBV, CMV and Polyoma BK

3.2 Inclusion Criteria

- ✓ Age 15-75 years at the time of screening.
- ✓ Biopsy proven cABMR with TG on biopsy as defined by Banff 2015 and DSA + at time of biopsy
- ✓ Subject/Parent/Guardian must be able to understand and provide informed consent.
- ✓ Pneumococcal vaccinated
- ✓ Negative tuberculin ppd result or negative Quantiferon TB gold

3.3 Exclusion Criteria

- ✓ Multi-organ transplant (e.g. kidney and pancreas)
- ✓ eGFR < 30 mL/min/1.73m^2
- ✓ Advanced Transplant Glomerulopathy (CG3) or advanced IFTA (ct or ci scores ≥3)
- ✓ Previous allergic reactions to monoclonal antibodies.
- ✓ Lactating or pregnant females.
- ✓ Women of child-bearing age and male partners of women of child-bearing age who are not willing or able to practice FDA-approved forms of contraception during study and for 5 months after last dose.
- ✓ HIV-positive subjects.
- ✓ Subjects who test positive for HBV by HBVeAg/DNA or HCV infection [positive Anti-HCV (EIA) and confirmatory HCV RIBA].

- ✓ Subjects with latent or active TB. Subjects must have negative Quantiferon TB gold test result.
- ✓ Recent recipients of any licensed or investigational live attenuated vaccine(s) within two months of the screening visit (including but not limited to any of the following:
    - Adenovirus [Adenovirus vaccine live oral type 7]
    - Varicella [Varivax]
    - Hepatitis A [VAQTA]
    - Rotavirus [Rotashield]
    - Yellow fever [Y-F-Vax]
    - Measles and mumps [Measles and mumps virus vaccine live]
    - Measles, mumps, and rubella vaccine [M-M-R-II]
    - Sabin oral polio vaccine
    - Rabies vaccines [IMOVAX Rabies I.D., RabAvert])
- ✓ A significantly abnormal general serum screening lab result defined as a WBC < 3.0 X 10^3/ml, a Hgb < 8.0 g/dL, a platelet count < 100 X 10^3/ml, an SGOT or SGPT > 3X upper limit normal
- ✓ Individuals deemed unable to comply with the protocol.
- ✓ Subjects with active CMV or EBV infection as defined by CMV-specific serology (IgG or IgM) and confirmed by quantitative PCR with or without a compatible illness (Quantitative PCR cut off defined as having > 50 copies of CMV or EBV DNA/PCR)
- ✓ Use of investigational agents within 4 weeks of participation.
- ✓ History or active Inflammatory Bowel Disease or Diverticular Disease or gastrointestinal perforation
- ✓ Recent infection (within past 6 weeks of screening) requiring any antibiotic use (oral, parenteral or topical).
- ✓ Present or previous (within 5 years) malignancy except for basal cell carcinoma, fully excised squamous cell carcinoma of the skin or non-recurrent (within 5 years) cervical carcinoma-in-situ.

4. Study Design & Methods

Background: This is a single center, Phase I/II, open label single-arm exploratory study. The trial will primarily examine the safety and tolerability of clazakizumab given after the diagnosis of cABMR in 10 subjects (15-75yrs) who exhibit DSAs to their donor. Patients entered will also have been diagnosed with cABMR + TG post-transplant based on Banff 2015 criteria. Patients are required to have a eGFR ≥ 30 mL/min/1.73m^2 as calculated by the MDRD equation (Schwartz equation will be used to estimate CrCl for patients under 18 years of age) at entry. All patients will be recruited from the renal transplant program at Cedars-Sinai Medical Center. Once cABMR is diagnosed, donor-specific anti-HLA antibodies will be assessed (DSA) which are associated with cABMR and/or graft loss. DSA will be detected using solid phase assay systems currently utilized at the Cedars-Sinai Medical Center HLA Laboratory (Dr. Xiaohai Zhang Director, Phone: 310-423-4979)[41]. These anti-HLA antibodies may result naturally or from previous pregnancy, transfusions, or prior transplants. Patients treated with clazakizumab for cABMR will have labs for DSAs, and other monitoring labs as well as immunologic studies as outlined (See Study Protocol in Appendix A). In addition to the standard post-transplant immunosuppressive protocol, patients with cABMR will receive clazakizumab 25mg SC given every 4 weeks (30 days) for a total of 6 doses. If no safety/tolerability/efficacy issues are observed after the initial dose, patients will continue the protocol as outlined (see Appendix A). A protocol biopsy will be performed after the 6th and after the 12th doses of clazakizumab (as mentioned before) to assess the allograft for evidence of cABMR/ABMR, including C4d staining and TG using Banff 2015 criteria[14]. Banff scoring will be compared between the index and protocol biopsy after cessation of therapy. Patients who have evidence of persistent allograft dysfunction may have non-protocol biopsies for cause. After completion of the clazakizumab therapy, patients will be followed up to assess allograft function and ABMR episodes as well as DSAs. The protocol is summarized in figure 14 below. Monitoring of T_{reg}, T_{h17}, T_{fh} and plasmablast as well as IL-6 and CRP level will be performed in the Transplant Immunology Lab at Cedars-Sinai Medical Center at select time points (Dr. Mieko Toyoda Director, Phone: 310-423-8282).

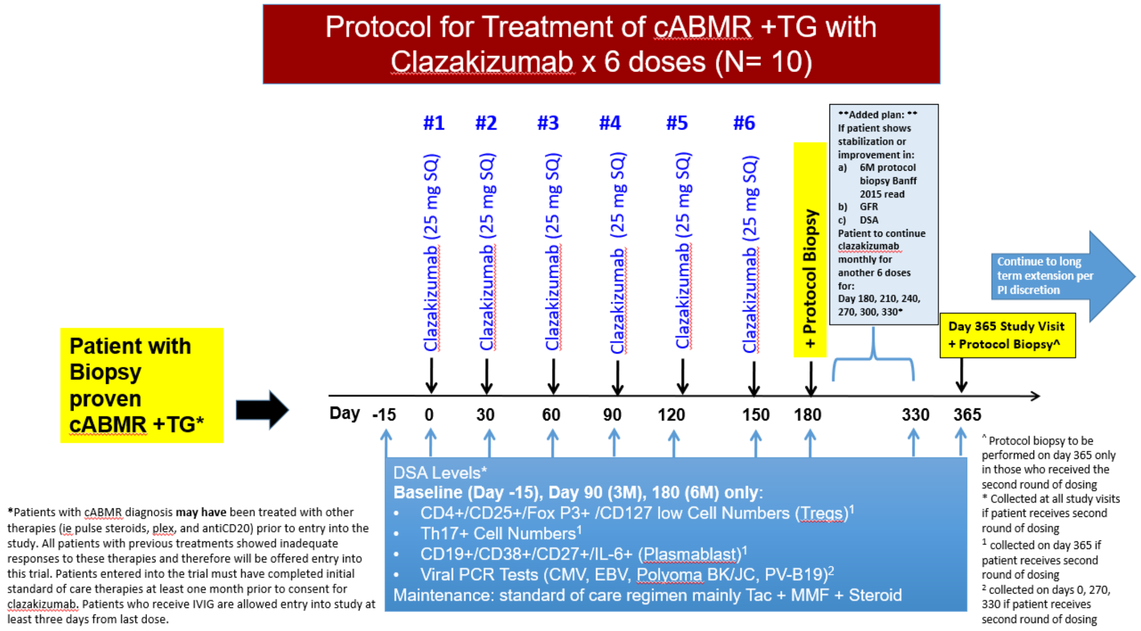

Figure 14: This figure shows the protocol designed to investigate the safety and efficacy of clazakizumab in treating cABMR + TG. The study is open label, single center one-arm study that will enter patients diagnosed with cABMR + TG by renal biopsy and who have eGFR of > 30cc/min at time of diagnosis. Entered patients will receive clazakizumab 25mg every 4 weeks (30 days) for a total of 6 doses. If patients show improvement (ie: eGFR/DSA/biopsy/PI confirmation) they will continue monthly clazakizumab for an additional 6 months and will have a second biopsy at 12 month protocol. Immunologic and viral monitoring labs will be performed as indicated. For those continuing in the long term extension, the day 365 visit will be followed by visits every 4- 8 weeks.

The subjects will be followed to determine if the use of clazakizumab for treatment of cABMR in this high-risk transplant population is safe and without infectious risks. In addition, we will determine the effects of clazakizumab treatment on renal biopsy assessments performed at 6 months. Assessments of renal function, donor specific antibody, and Banff 2015 biopsy scores will be evaluated at that time. If improvement or stabilization observed, clazakizumab will be resumed monthly x 6 doses (starting day 180 to day 330) and last study visit will be day 365 with biopsy for those who are not continuing to the long term extension. For those continuing in the long term extension, the day 365 visit will be followed by visits every 4- 8 weeks, per the schedule in Appendix E. We will assess the transplanted patients to determine the number who sustain a viable and functioning kidney allograft as well. In the event a patient does not show improvement after receiving 6 doses of clazakizumab, no further treatment will be given and the patient will return at Day 365 for a final study visit. All subjects will be evaluated on an intent-to-treat basis. The subject accrual rate will be limited to 2 patients for the initial dosing appointment. Once the first 2 patients have received their first injection, 2-4 patients may be screened per month until all 10 patients are enrolled. Repeat laboratories will be performed at the completion of clazakizumab therapy to determine effect on levels and correlation with any potential events. A detailed analysis of the study is discussed below.

A long term dosing option at conclusion of 12 months (after Study Day 365 visit) For those patients who complete the 12 month protocol, patients may continue to receive clazakizumab 25 mg subQ every 4- 8 weeks long term, per PI discretion. Please see Appendix E for long term visit schedule. Biopsies during the long term extension will be conducted at 12 months per PI discretion or for cause (suspected rejection). The long term extension of this study is planned for a duration of 3 years.

4.1 Study Analysis

This single-center, single-arm, Phase I/II, trial is designed to examine the safety, tolerability and limited efficacy of human clazakizumab (25mg SC Q4W) given as per protocol (Appendix A) in 10 subjects who are DSA + and have biopsy proven cABMR + TG. Patients considered for this study may have been treated with high-dose IVIG + rituximab and/or plasmapheresis[3,6,7] In addition, some patients with early, severe ABMR may have been treated with Eculizumab[42].

4.2 Defining ABMR

For purposes of this investigation, antibody-mediated rejection (ABMR) is defined as follows:

- ✓ Deterioration of allograft function in a transplant recipient measured by eGFR (defined as a eGFR $\geq$ 30cc/min reduction from baseline).
- ✓ Association with the presence of DSA (usually increasing in strength) measured by luminex techniques.
- ✓ Biopsy evidence of cABMR and TG by biopsy by Banff 2015 criteria.

4.3 Treatment of Allograft Rejection Episodes During the Study

Biopsy-proven rejection episodes that occur during the study are treated with "pulse" methylprednisolone (10mg/kg/day, max 1000mg for >100kg for 3 days) and anti-thymocyte globulin (1.5mg/kg daily X 4) for cell-mediated rejection episodes that are unresponsive to pulse steroids. Patients experiencing recurrent ABMR episodes after study drug treatment, will initially receive pulse methylprednisolone (10mg/kg/day, max 1000mg for >100kg) IV daily x 3 doses then, depending on severity, IVIG 10% solution 2gm/kg (max 140g for >70kg) IV X1 dose followed by rituximab (375mg/m2 rounded to the nearest 100mg) IV X1 dose three to five days after last IVIG dose. In cases where rapid deterioration of allograft function is seen and/or thrombotic microangiopathy diagnosed, the patient will receive plasma exchange X 3-5 sessions followed by anti-C5 (Eculizumab®) IV weekly X4 weeks (1200mg week #1 followed by 900mg/ weekly for 3 additional weeks). Efficacy of therapy will be assessed by determining renal function improvement, monitoring DSA responses and repeat allograft biopsies, if needed.

4.4 Monitoring for AE/SAEs Post-Transplant in HS Patients

Adverse events (AEs) and serious adverse events will be monitored post-ABMR treatment with clazakizumab. These include careful attention to infectious complications potentially associated with clazakizumab therapy.

Infectious complications associated with IVIG + rituximab desensitization and alemtuzumab induction therapy followed by maintenance therapy with tacrolimus, MMF and prednisone have been assessed by our group. Briefly, we evaluated 170 patients who were desensitized with IVIG + rituximab followed by alemtuzumab induction and maintenance therapy with tacrolimus, MMF and steroids. This was compared to a concomitant group of non-sensitized, low-risk transplants (N=191) who did not receive IVIG, rituximab or alemtuzumab (induction with IL-2R blockers). A careful analysis of all infections and serious infections that occurred over the next 4 years was compiled and is shown in Figure 15A &15B. Briefly, these data show that the use of this desensitization protocol followed with alemtuzumab induction does not increase the risk for common or serious infections post-transplant compared to a low risk group of patients. Serious infections were defined as any viral infection and fungal or bacterial infections requiring i.v. antibiotics or hospitalizations[41]. Thus risk for infections in the study group (clazakizumab) after ABMR treatment will likely be similar and comparable to non-sensitized patients. All patients entered into this study are required to be vaccinated for *Streptococcus pneumoniae*.

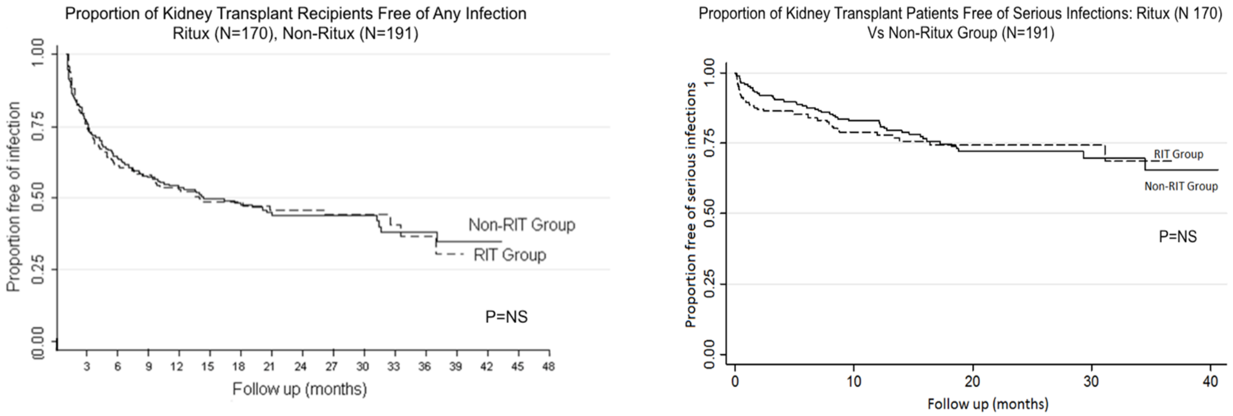

15A & 15B: This figure shows the proportion of kidney transplant recipients who developed infections (all infections 15A, or serious infections 15B) post-transplant. Patients were in the low-risk group (non-Ritux #191) who did not receive IVIG/ritux or alemtuzumab or (Ritux #171) who received desensitization with rituximab, IVIG followed by transplantation. Follow up for up to 45 months shows that infection rates are similar. The commonest infection was urinary tract infection in both groups. The incidence of CMV was greater in the non-Ritux group while there were more BK infections in the Ritux group. No patients in the Ritux group developed PTLD or PML while 2 patients in the non-Ritux group developed PTLD[43].

More recent safety data on viral infections in more than 400 highly HLA sensitized patients treated with IVIG + rituximab and Campath-1H induction therapy were compared to ~600 patients who were non-sensitized and did not receive desensitization. The data are shown below:

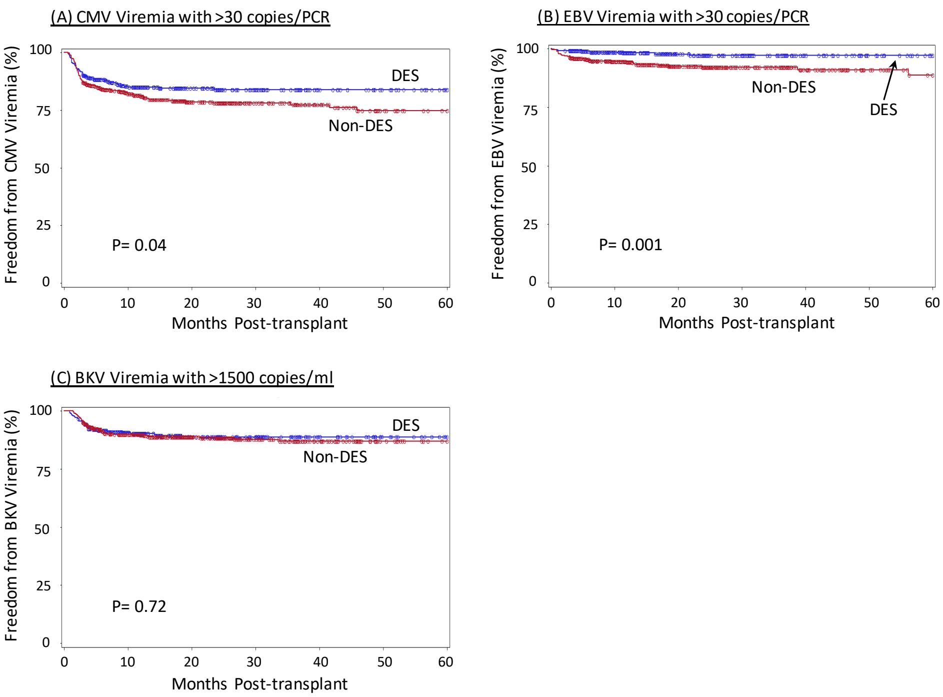

Figure 16: The data shown above show the viral PCR positivity for CMV, EBV and polyma BK virus in 400 desensitized patients compared with 600 standard kidney transplant recipients. Of interest are the significantly lower incidence of EBV and CMV viremia in desensitized individuals compared with normal non-sensitized transplant recipients. No differences were seen between the highly-HLA sensitized patients and non-sensitized patients in incidence of BK

viremia. Thus, no significant risk for viremia is seen, in fact, there appears to be a benefical effect of DES on reducing incidence of CMV and EBV viremia.

4.5 Safety Reporting of Adverse Events

Safety Reporting of Adverse Events

Assessment of Safety

Specification of Safety Variables

Safety assessments will consist of monitoring and reporting all adverse events (AEs) and serious adverse events (SAEs) , all events of death, and any study specific issue of concern.

Adverse Events

An AE is any unfavorable and unintended sign, symptom, or disease temporally associated with the use of an investigational medicinal product (IMP) or other protocol-imposed intervention, regardless of attribution.

This includes the following:

- AEs not previously observed in the subject that emerge during the protocol-specified AE reporting period, including signs or symptoms associated with Clazakizumab infusion that were not present prior to the AE reporting period.
- Complications that occur as a result of protocol-mandated interventions (e.g., renal protocol biopsy).
- If applicable, AEs that occur prior to assignment of study treatment associated with medication washout, no treatment run-in, or other protocol-mandated intervention.
- Preexisting medical conditions (other than the condition being studied) judged by the investigator to have worsened in severity or frequency or changed in character during the protocol-specified AE reporting period.

Serious Adverse Events

An AE should be classified as an SAE if the following criteria are met:

- It results in death (i.e., the AE actually causes or leads to death).
- It is life threatening (i.e., the AE, in the view of the investigator, places the subject at immediate risk of death. It does not include an AE that, had it occurred in a more severe form, might have caused death.).
- It requires or prolongs inpatient hospitalization.
- It results in persistent or significant disability/incapacity (i.e., the AE results in substantial disruption of the subject's ability to conduct normal life functions).
- It results in a congenital anomaly/birth defect in a neonate/infant born to a mother exposed to the IMP.
- It is considered a significant medical event by the investigator based on medical judgment (e.g., may jeopardize the subject or may require medical/surgical intervention to prevent one of the outcomes listed above).

Methods and Timing for Assessing AND Recording Safety variables

The investigator is responsible for ensuring that all AEs and SAEs that are observed or reported during the study, are collected and reported to the FDA, appropriate IRB(s), and CSL Behring LLC. in accordance with CFR 312.32 (IND Safety Reports).

Adverse Event Reporting Period
The study period during which all AEs and SAEs must be reported begins after informed consent is obtained and initiation of study treatment and ends 150 days after the last dose of study drug~~visit~~.

4.5.1 Reporting of Serious Adverse Events Associated with Clazakizumab

Any Serious Adverse Event (SAE)/Serious Adverse Reaction (SAR)/Special Situation that occurs during the course of the STUDY and is associated with the use of a CSLB study drug must be forwarded within **one (1) working day** from Date of First Receipt to CSLB by e-mail or fax (see Table) irrespective of whether it is assessed to be related to this product.

The exchange of all SAE/SAR/AR case report information shall be in English language by using a Case Report Form agreed on by CSLB.

CSLB shall acknowledge receipt within **two (2) working days**. Should the INSTITUTION not receive a confirmation of the case receipt within the **two (2) working days**, the INSTITUTION shall request confirmation that the report has reached CSLB.

The INSTITUTION should cooperate with CSLB and provide missing information in a timely manner as well as responses to queries until the event has resolved, or, in case of permanent impairment, until stabilized. Follow-up information obtained by the INSTITUTION should be handled accordingly, and forwarded as per the timelines stated above.

Contact for Exchange of SAE/SAR/AR *resp*. SUSAR/Pregnancy/Special Situation Information:

Name	Helen Sborlini (Case Reports)<br>Samantha Gan (AE-Reconciliation)
Title	Case Management Team Lead, CSLB-GCSP King of Prussia US<br>ICSR Compliance and Reconciliation Lead, CSLB-GCSP Parkville AU
e-mail	AE reporting: adverse.events.global@CSL Behring.com<br>Global AE reconciliation email:<br>Global.Reconciliation@csl.com.au
Fax	+1 610-878-4487

Contact for other Safety Issues regarding the PRODUCT:

Name	David Gao
Title	Director, Clinical Safety Physician, Immunology<br>Global Clinical Safety and Pharmacovigilance
e-mail	Zhiyong.Gao@cslbehring.com
Phone	+1 610-290-7659 (for AE Reporting only)

Contact for Study Report distribution (refer to section 1.3):
email subject 'Report from Clazakizumab IIT, please distribute to responsible CSP/CSS and CM'

Name	Global Periodic Reports Office
e-mail	PeriodicReports.GCSP@cslbehring.com

SAE Reporting	Contact Information
Study Coordination Center/Principal Investigator: Stanley C. Jordan, M.D.	Tel: 310-423-2641<br>Fax: 310-423-6369
IRB Contact information and fax #<br>Keren Dunn<br>Cedars-Sinai Medical Center<br>Director, Research Compliance	Tel: 310-423-3783<br>Fax: 310-423-4195

AND:

MedWatch 3500A Reporting Guidelines:

In addition to completing appropriate patient demographic and suspect medication information, the report should include the following information within the Event Description (section 5) of the MedWatch 3500A form:

- Treatment regimen (dosing frequency, combination therapy)
- Protocol description (and number, if assigned)
- Description of event, severity, treatment, and outcome if known
- Supportive laboratory results and diagnostics
- Investigator's assessment of the relationship of the adverse event to each investigational product and suspect medication

Follow-up information:
Additional information may be added to a previously submitted report by any of the following methods:

- Adding to the original MedWatch 3500A report and submitting it as follow-up
- Adding supplemental summary information and submitting it as follow-up with the original MedWatch 3500A form.

- Summarizing new information and faxing it with a cover letter including subject identifiers (i.e. D.O.B. initial, subject number), protocol description and number, if assigned, suspect drug, brief adverse event description, and notation that additional or follow-up information is being submitted (The patient identifiers are important so that the new information is added to the correct initial report)

Occasionally CSL Behring may contact the reporter for additional information, clarification, or current status of the subject for whom and adverse event was reported. For questions regarding SAE reporting, you may contact the CSL Behring Drug Safety representative noted above.

With completion of the STUDY, the INSTITUTION shall provide to CSLB via email or fax (see APPENDIX 01) the final/clinical study report within three months after completion of Study conduct or termination of this agreement.. Any interim report is to be provided by the INSTITUTION has to be forwarded on a bi-annual basis.

In addition, the INSTITUTION shall provide a line listing/table including the causality assessment for each SAE/SAR/AR/AE/Special Situation within **five (5) working days** after closure of the database. The PARTIES will agree on the event-level detail prior to forwarding to CSLB.

Study Drug Relationship:

The investigator will determine which events are associated with the use of the study drugs. The causality assessment is the determination of whether there exists a reasonable possibility that the Study treatment caused or contributed to an adverse event:

- <u>Not related:</u> Temporal relationship to Study treatment administration is missing or implausible, or there is evidence of another cause.

- <u>Possibly related:</u> Reasonable time sequence to administration of Study treatment, but the event could also be explained by concurrent disease of other drugs or chemicals. Information on drug withdrawal may be lacking or unclear.

- <u>Definitely related</u>**:** Plausible time relationship to Study treatment administration; event cannot be explained by concurrent disease or other drugs or chemicals. The response to withdrawal of the drug (dechallenge) should be clinically plausible. The event must be definitive pharmacologically or phenomenologically, using a satisfactory re-challenge procedure if necessary.

4.5.2

Pregnancy Reporting

Should a patient or female partner of a male under treatment with a CSLB product become pregnant during the course of the STUDY, this information must be forwarded to CSLB within **ten (10) calendar days**. The INSTITUTION shall follow-up until outcome of pregnancy is known and potential safety concerns have been clarified. As soon as outcome of pregnancy is available to the INSTITUTION this information has to be provided to CSLB within **one (1) working day.**

In case of a pregnancy in the female partner of a male subject, the Investigator should obtain informed consent of the pregnant partner prior to monitoring of the pregnancy.

4.6 Infection Prophylaxis Protocols and Viral Monitoring Post-Transplant

All study patients, regardless of their cytomegalovirus (CMV) status, receive IV ganciclovir while inpatients and valganciclovir as outpatients for 6 months post kidney transplant, with dose adjustments for renal function. Fungal prophylaxis was accomplished with fluconazole 100 mg daily for 1 month post transplant. Pneumocystis jirovecii pneumonia and bacterial prophylaxis is accomplished with trimethoprim 80 mg and sulfamethoxazole 400 mg daily for 12 months post transplant. No additional prophylaxis will be needed for patients who are enrolled in this trial more than one year from transplant.

Viral polymerase chain reaction assays for CMV, Epstein Barr virus, Parvovirus B-19, Polyoma virus BK and JC will be performed on study patients monthly for 6 months post-transplantation. Methodologies used for monitoring viral replication have been described previously [25].

5 Dosing of Clazakizumab

For antibody mediated rejection: Clazakizumab will be administered at a dose of 25mg/SC every 4 weeks post pulse methylprednisolone, plasmapheresis, and high dose IVIG. . If the patient proceeds to the LTE, they will receive clazakizumab 25 mg SC Q8W.
Product Description, Storage and Administration Instructions

Clazakizumab will be provided by CSL Behring LLC.

Generic name:	Clazakizumab
Active ingredient:	Genetically engineered humanized anti-IL-6 mAb
Strength:	25 mg/mL or 12.5 mg/mL
Excipients:	L-histidine, L-histidine monohydrochloride, sorbitol, polysorbate-80, and water for injection
Appearance:	Clear to slightly opaque, colorless to dark yellow-colored solution
Dosage form:	Single-dose vials (25 mg/mL or 12.5 mg/mL) for injection.

Manufacturer: Ajinomoto Althea, San Diego CA
Patheon Manufacturing Services LLC, Greenville, NC
Clazakizumab vials should be stored at ≤-20°C (-4°F) with protection from light.

The drug product will be administered undiluted at a concentration of 25 mg/mL or 12.5 mg/mL.

Prepared syringes may be stored for up to 24 hours in a refrigerator, 2°-8°C (36°-46°F), and up to 4 hours of the 24 hours may be at room temperature, 15°-25°C (59°-77°F). The prepared syringes should be protected from light.

Prior to administration, clazakizumab should reach room temperature by storing unrefrigerated for 30 to 60 minutes before use.

IP requests should be sent to: CentralGroup.CTS@cslbehring.com

5.1 Storage and Handling

Clazakizumab will not be used after the expiry date (EXP) shown on the kit or vial.

5.1.1 Clazakizumab Overdose

There are no specific antidotes or measures to take in the event of an overdose of clazakizumab injection. Subjects should be treated with the appropriate supportive care.

5.2 Dose Modification/Toxicity Management

A number of measures will be taken to ensure the safety of patients participating in this study. These measures will be addressed through exclusion criteria (see Section 4.2) and routine monitoring as follows:

Patients enrolled in this study will be evaluated clinically and with standard laboratory tests before and during their participation in this study. Safety evaluations will consist of medical interviews, recording of adverse events, physical examinations, blood pressure, and laboratory measurements. Subjects will be evaluated for adverse events (all grades), serious adverse events, and adverse events requiring study drug interruption or discontinuation at each study visit for the duration of their participation in the study.

5.3 Adverse Drug Reactions

Opportunistic Infections and Serious Infections

Physicians should exercise caution when considering the use of clazakizumab in patients with a history of recurring infection or with underlying conditions (eg, diabetes) which may predispose patients to infections. Clazakizumab should not be administered in patients with active infection. The effects of clazakizumab on CRP, neutrophils, and the signs and symptoms of infection should be considered when evaluating a patient for a potential infection.

Vigilance for timely detection of serious infection is recommended for patients receiving biologic agents for treatment of moderate to severe RA as signs and symptoms of acute inflammation may be lessened due to suppression of the acute phase reaction. Patients must be instructed to contact their physician immediately when any symptoms suggesting infection appear, in order to assure rapid evaluation and appropriate treatment.

If a patient develops a serious infection, administration of clazakizumab is to be interrupted until the infection is controlled. The clinician should consider the benefit-risk before resuming treatment with clazakizumab.

Gastrointestinal Perforations

Timely diagnosis and appropriate treatment may reduce the potential for complications of diverticulitis and thus reduce the risk of GI perforations. Therefore, patients should be made aware of the symptomatology potentially indicative of diverticular disease, and they should be instructed to alert their healthcare provider as soon as possible if these symptoms arise. In patients with a history of symptomatic diverticulosis, diverticulitis or chronic ulcerative lower GI disease such as Crohn’s disease, ulcerative colitis or other chronic lower GI conditions that might predispose to perforations, the clinician should consider the benefit-risk before using clazakizumab. Discontinuation of clazakizumab is recommended for patients who develop GI perforations.

Demyelinating Disorders

The impact of treatment with clazakizumab on demyelinating disorders is not known; events were rarely reported. Patients should be closely monitored for signs and symptoms potentially indicative of central demyelinating disorders. Physicians should exercise caution in considering the use of clazkizumab in patients with pre-existing or recent onset demyelinating disorders. Treatment with clazakizumab should be interrupted during assessment of a potential demyelination event and only resumed if the benefit of continuing study drug is favorable.

Hematologic Abnormalities and Bleeding Events

Decreases in neutrophil and platelet counts have been observed following treatment with clazakizumab in combination with MTX. In addition, there may be an increased risk of neutropenia in patients who have previously been treated with a TNF antagonist.

For patients with concomitant medications associated with hematologic toxicity, the reduction or interruption of the suspected medication is recommended prior to modifying clazakizumab.

Table 2: Neutropenia Risk Mitigation

ANC (cells/mm^3)	Action
> 1000	Maintain dose.
500 – 1000	Interrupt clazakizumab dosing.<br><br>When ANC increases to > 1000, resume clazakizumab 25mg SQ monthly
< 500	Discontinue clazakizumab.
ANC = absolute neutrophil count	

Table 3: Thrombocytopenia Risk Mitigation

Platelet count (cells/mm^3)	Action
> 100,000	Maintain dose.
50,000 – 100,000	Interrupt clazakizumab dosing.<br><br>When platelet count increases to > 100,000, resume Clazakizumab at 25mg SQ monthly
< 50,000	Discontinue clazakizumab.

Elevated Liver Enzymes and Hepatic Events

Elevations in ALT and AST have been observed during treatment with the study medications

Table 4:

Lab Value	Action
> 1 to 3x ULN	Dose modify concomitant transplant immunosuppressive drugs if appropriate<br><br>For persistent increases in this range, interrupt clazakizumab until ALT/AST have normalized<br><br>Restart with 25mg SQ monthly as clinically appropriate
> 3 to 5x ULN<br><br>(confirmed by repeat testing)	Interrupt clazakizumab dosing until < 3x ULN and follow<br><br>recommendations above for >1 to 3x ULN<br><br>For persistent increases > 3x ULN, discontinue Clazakizumab<br><br>Discontinue clazakizumab if total bilirubin >2 x ULN
> 5x ULN	Discontinue clazakizumab

Cardiovascular Events and Elevated Lipids

Patients with RA have an increased risk for cardiovascular disorders, therefore, risk factors for cardiovascular disease (eg, hypertension, hyperlipidemia) should be managed as part of their standard of care. See section on Drug Interactions.

For patients with LDL cholesterol ≥160 mg/dL, it is strongly recommended that investigators advise therapeutic lifestyle changes that may include initiation lipid-lowering agents. Lipid-lowering agents should also be considered for patients with lower LDL cholesterol levels as part of their therapeutic lifestyle changes depending on their overall risk as defined in NCEP ATP III or other national guidelines.

Malignancies

The impact of immunosuppression on the development of malignancies is not known, however an increased rate of some malignancies, notably lymphoma, has been observed in RA patients. Although no imbalance of malignancies was observed in clinical trials of clazakizumab, malignancies have been identified as a concern for other biologics. It is recognized that identification of such events in clazakizumab-treated patients may require a longer period of surveillance. Clazakizumab should be discontinued in patients with malignancies (with the exception of local basal or squamous cell carcinoma of the skin that is completely excised with free margins).

Hypersensitivity or Anaphylaxis:

An infusion/dose reaction is defined as an adverse event occurring during and within 24 hours after the infusion or subcutaneous injection of clazakizumab. This may include hypersensitivity reactions or anaphylactic reactions.

Signs of a possible hypersensitivity reaction include but are not limited to:

- fever, chills, pruritus, urticaria, angioedema, and skin rash.
- cardiopulmonary reactions, including chest pain, dyspnea, hypotension or hypertension.

Healthcare professionals administering clazakizumab should be trained in the appropriate administrative procedures, be able to recognize the symptoms associated with potential anaphylactic or hypersensitivity reactions, and have the appropriate medication available for immediate use in case of anaphylaxis or hypersensitivity reaction during or after administration of clazakizumab. Healthcare professionals should also instruct patients to seek medical attention if they experience symptoms of a hypersensitivity reaction outside of the clinic.

If a patient has symptoms of anaphylaxis or serious hypersensitivity, or requires an interruption of the study drug because of symptoms of anaphylaxis or hypersensitivity, administration of clazakizumab must be discontinued permanently. The patient should be treated according to the standard of care for management of the hypersensitivity reaction. A blood sample for the presence of anti-clazakizumab antibodies should be obtained.

Clazakizumab should not be administered to subjects who have had any previous allergic reactions to monoclonal antibodies. To date, no infusion reactions have been associated with clazakizumab administered by IV infusion. Injection site reactions have been reported with SC administration. Reactions have been mild or moderate and have resolved without treatment. Both allergic reactions and injection site reactions should be treated with standard of care. Subjects who have developed significant allergic reaction to study drugs should not be re-challenged.

Viral Reactivation

Though rarely reported within the clazakizumab program due to exclusion criteria at study entry, reactivation of viral and other serious infections (e.g. EBV or TB) has been observed with biologic therapies.

Drug Interaction

The formation of CYP450 enzymes may be suppressed by increased levels of cytokines (eg, IL-6) during chronic inflammation. Therefore, it is expected that for molecules that antagonize cytokine activity, such as clazakizumab, the formation of CYP450 enzymes could be normalized. When starting or stopping therapy with clazakizumab, patients taking medications which are individually dose-adjusted and metabolized via CYP450, 3A4, 1A2, or 2C9 (e.g. atorvastatin, calcium channel blockers, theophylline, warfarin, phenytoin, cyclosporine, or benzodiazepines) should be monitored as doses may need to be adjusted to maintain their therapeutic effect. Given its long elimination half-life (t1/2), the effect of clazakizumab on CYP450 enzyme activity may persist for several weeks after stopping therapy.

Pregnancies and Women of Child Bearing Potential

There are no adequate well-controlled studies in pregnant or lactating women. In nonclinical studies, an increase in the number of monkeys with retention of the placenta at parturition was observed at clazakizumab doses corresponding to 11 and 110 times the planned human dose of 50 mg. In 3 of the 5 monkeys with retained placentas, the resulting excessive uterine hemorrhage led to moribund status in the mothers.

Three pregnancies have been reported to date in subjects taking clazakizumab. The outcomes included one spontaneous abortion (outcome unknown for the other 2 pregnancies).

All subjects of child bearing potential being treated with clazakizumab (and their partners) must be informed of this risk, and use highly effective birth control. Administration of clazakizumab may decrease the efficacy of hormonal oral contraceptives.

Under no circumstances shall clazakizumab injection be administered to women known to be pregnant or lactating.

6 Therapy Stopping Points

As indicated previously, the study will be halted and re-evaluated by the Data and Safety Monitoring Board (DSMB) if any patient in the study group develops SAEs or evidence of severe infusion related or infectious complications. In addition the study will be reevaluated if a patient develops worsening ABMR while in treatment, possibly indicating lack of efficacy of clazakizumab (see appendix C).

7 Statistical Analysis

Due to the exploratory nature of this study that involves safety endpoints only and the small sample size that is not powered for efficacy end points, our primary objective will be to assess safety end points and limited efficacy. Most important will be tolerability in the ESRD population and determination of the effects of clazakizumab treatment on circulating DSAs as well as the ability to improve outcomes of patients with cABMR and TG. We will however plan to assess biopsies 6M post-transplantation in patients desensitized with clazakizumab treatment to determine if there are durable effects in prevention of ABMR both acute and chronic.

8 References

1. Colvin RB. Antibody-mediated renal allograft rejection: diagnosis and pathogenesis. J Am Soc Nephrol 2007;18:1046-56.
2. Djamali A, Kaufman DB, Ellis TM, Zhong W, Matas A, Samaniego M. Diagnosis and management of antibody-mediated rejection: current status and novel approaches. Am J Transplant 2014;14:255-71.
3. Jordan SC, Tyan D, Stablein D, et al. Evaluation of intravenous immunoglobulin as an agent to lower allosensitization and improve transplantation in highly sensitized adult patients with end-stage renal disease: report of the NIH IG02 trial. J Am Soc Nephrol 2004;15:3256-62.
4. Marfo K, Lu A, Ling M, Akalin E. Desensitization protocols and their outcome. Clin J Am Soc Nephrol 2011;6:922-36.
5. Montgomery RA, Lonze BE, King KE, et al. Desensitization in HLA-incompatible kidney recipients and survival. N Engl J Med 2011;365:318-26.
6. Vo AA, Lukovsky M, Toyoda M, et al. Rituximab and intravenous immune globulin for desensitization during renal transplantation. N Engl J Med 2008;359:242-51.
7. Vo AA, Petrozzino J, Yeung K, et al. Efficacy, outcomes, and cost-effectiveness of desensitization using IVIG and rituximab. Transplantation 2013;95:852-8.
8. Sellarés J, de Freitas DG, Mengel M, et al. Understanding the causes of kidney transplant failure: the dominant role of antibody-mediated rejection and nonadherence. Am J Transplant 2012;12:388-99.
9. Loupy A, Hill GS, Jordan SC. The impact of donor-specific anti-HLA antibodies on late kidney allograft failure. Nat Rev Nephrol 2012;8:348-57.
10. Solez K, Colvin RB, Racusen LC, et al. Banff '05 Meeting Report: differential diagnosis of chronic allograft injury and elimination of chronic allograft nephropathy ('CAN'). Am J Transplant 2007;7:518-26.
11. Gloor J, Cosio F, Lager DJ, Stegall MD. The spectrum of antibody-mediated renal allograft injury: implications for treatment. Am J Transplant 2008;8:1367-73.
12. Lefaucheur C, Loupy A, Vernerey D, et al. Antibody-mediated vascular rejection of kidney allografts: a population-based study. Lancet 2013;381:313-9.
13. Jordan SC, Reinsmoen N, Peng A, et al. Advances in diagnosing and managing antibody-mediated rejection. Pediatr Nephrol 2010;25:2035-45; quiz 45-8.
14. Haas M, Sis B, Racusen LC, et al. Banff 2013 meeting report: inclusion of c4d-negative antibody-mediated rejection and antibody-associated arterial lesions. Am J Transplant 2014;14:272-83.
15. Tanaka T, Kishimoto T. The biology and medical implications of interleukin-6. Cancer Immunol Res 2014;2:288-94.
16. Tanaka T, Narazaki M, Kishimoto T. IL-6 in inflammation, immunity, and disease. Cold Spring Harb Perspect Biol 2014;6:a016295.
17. Kim I, Wu G, Chai NN, Klein AS, Jordan S. Anti-interleukin 6 receptor antibodies attenuate antibody recall responses in a mouse model of allosensitization. Transplantation 2014;98:1262-70.
18. Hunter CA, Jones SA. IL-6 as a keystone cytokine in health and disease. Nat Immunol 2015;16:448-57.
19. Jordan SC, Choi J, Kim I, et al. Interleukin 6 (IL-6) a Cytokine Critical to Mediation of Inflammation, AutoImmunity and Allograft Rejection: Therapeutic Implications of IL-6 Receptor Blockade. Transplantation 2016.
20. Redfield RR, Ellis TM, Zhong W, et al. Current outcomes of chronic active antibody mediated rejection - A large single center retrospective review using the updated BANFF 2013 criteria. Hum Immunol 2016;77:346-52.
21. Wiebe C, Gibson IW, Blydt-Hansen TD, et al. Rates and determinants of progression to graft failure in kidney allograft recipients with de novo donor-specific antibody. Am J Transplant 2015;15:2921-30.
22. Kim I, Wu G, Chai NN, Klein AS, Jordan S. Anti-Interleukin 6 Receptor Antibodies Attenuate Antibody Recall Responses in a Mouse Model of Allosensitization. Transplantation 2014.
23. Wu G, Chai N, Kim I, Klein AS, Jordan SC. Monoclonal anti-interleukin-6 receptor antibody attenuates donor-specific antibody responses in a mouse model of allosensitization. Transpl Immunol 2013;28:138-43.
24. Shirota Y, Yarboro C, Fischer R, Pham TH, Lipsky P, Illei GG. Impact of anti-interleukin-6 receptor blockade on circulating T and B cell subsets in patients with systemic lupus erythematosus. Ann Rheum Dis 2013;72:118-28.

25. Illei GG, Shirota Y, Yarboro CH, et al. Tocilizumab in systemic lupus erythematosus: data on safety, preliminary efficacy, and impact on circulating plasma cells from an open-label phase I dosage-escalation study. Arthritis Rheum 2010;62:542-52.
26. Tanaka T, Kishimoto T. Targeting interleukin-6: all the way to treat autoimmune and inflammatory diseases. Int J Biol Sci 2012;8:1227-36.
27. Kang S, Tanaka T, Kishimoto T. Therapeutic uses of anti-interleukin-6 receptor antibody. Int Immunol 2015;27:21-9.
28. Wu G, Kim I, Chai N-n, et al. CTLA4Ig Differentially Suppresses *de novo* and Recall Alloantibody Responses in a Mouse Model of HLA.A2 Allosensitization. WTC 2014 Abstract2014.
29. Chavele KM, Merry E, Ehrenstein MR. Cutting edge: circulating plasmablasts induce the differentiation of human T follicular helper cells via IL-6 production. J Immunol 2015;194:2482-5.
30. Kikuchi J, Hashizume M, Kaneko Y, Yoshimoto K, Nishina N, Takeuchi T. Peripheral blood CD4(+)CD25(+)CD127(low) regulatory T cells are significantly increased by tocilizumab treatment in patients with rheumatoid arthritis: increase in regulatory T cells correlates with clinical response. Arthritis Res Ther 2015;17:10.
31. Tawara I, Koyama M, Liu C, et al. Interleukin-6 modulates graft-versus-host responses after experimental allogeneic bone marrow transplantation. Clin Cancer Res 2011;17:77-88.
32. Zhao X, Boenisch O, Yeung M, et al. Critical role of proinflammatory cytokine IL-6 in allograft rejection and tolerance. Am J Transplant 2012;12:90-101.
33. Vo AA, Choi J, Kim I, et al. A Phase I/II Trial of the Interleukin-6 Receptor Specific Humanized Monoclonal (Tocilizumab) + Intravenous Immunoglobulin in Difficult to Desensitize Patients. Transplantation 2015.
34. Aoyama A TM, Smith R-N, et al. Non-Human Primate Lung Allograft Survival is Prolonged by IL-6 Inhibition and ATG Treatment Possibly Through Expansion of Peripheral Regulatory T Cells. [Abstract]. Am J Transplant2016.
35. Vo AA, Choi J, Kim I, et al. A Phase I/II Trial of the Interleukin-6 Receptor-Specific Humanized Monoclonal (Tocilizumab) + Intravenous Immunoglobulin in Difficult to Desensitize Patients. Transplantation 2015;99:2356-63.
36. Aubert O, Loupy A, Hidalgo L, et al. Antibody-Mediated Rejection Due to Preexisting versus De Novo Donor-Specific Antibodies in Kidney Allograft Recipients. J Am Soc Nephrol 2017.
37. Fogal B, Yi T, Wang C, et al. Neutralizing IL-6 reduces human arterial allograft rejection by allowing emergence of CD161+ CD4+ regulatory T cells. J Immunol 2011;187:6268-80.
38. Loupy A, Lefaucheur C, Vernerey D, et al. Complement-binding anti-HLA antibodies and kidney-allograft survival. N Engl J Med 2013;369:1215-26.
39. Vo AA, Sinha A, Haas M, et al. Factors Predicting Risk for Antibody-Mediated Rejection and Graft Loss in Highly Human Leukocyte Antigen Sensitized Patients Transplanted After Desensitization. Transplantation 2015.
40. Choi J, Aubert O, Vo A, et al. Assessment of Tocilizumab (Anti-IL-6 Receptor Monoclonal) as a Potential Treatment for Chronic Antibody Mediated Rejection and Transplant Glomerulopathy in HLA Sensitized Renal Allograft Recipients. Am J Transplant 2017.
41. Reinsmoen NL, Lai CH, Vo A, et al. Acceptable donor-specific antibody levels allowing for successful deceased and living donor kidney transplantation after desensitization therapy. Transplantation 2008;86:820-5.
42. Stegall MD, Diwan T, Raghavaiah S, et al. Terminal complement inhibition decreases antibody-mediated rejection in sensitized renal transplant recipients. Am J Transplant 2011;11:2405-13.
43. Kahwaji J, Sinha A, Toyoda M, et al. Infectious complications in kidney-transplant recipients desensitized with rituximab and intravenous immunoglobulin. Clin J Am Soc Nephrol 2011;6:2894-900.

Appendix A Study/Protocol: A Phase I/II Trial to Evaluate the Safety and Tolerability of Clazakizumab® (Anti-IL-6 monoclonal) As an Agent to Eliminate Donor Specific HLA Antibodies (DSAs) and Improve Outcomes of Patients with Chronic & Active Antibody-Mediated Rejection (cABMR) Post-Kidney Transplantation

**Study visit**	Screening (-15 days)	Day 0 CLAZ#1	Day 30 ± 7d	Day 60 ± 7 days	Day 90 ± 7 days	Day 120 ± 7days	Day 150 ± 7days	Day 180 ± 30 days Biopsy[#] **	Day 210 ± 7 days**	Day 240 ± 7 days**	Day 270 ± 7 days**	Day 300 ± 7 days**	Day 330 ± 7 days**	Day 365 ± 30 days[5]
Medical History	X	X		X			X	X						X
Complete Physical Exam	X	X		X			X	X						X
Vital signs/weight	X	X	X	X	X	X	X	X	**X	**X	**X	**X	**X	X
12-lead ECG (SOC)+	X													
Chest X-Ray (SOC)+	X													
Safety laboratory tests (CBC with differential, CMP, UA)+^	X		X	X	X	X	X	X	**X	**X	**X	**X	**X	X
Review Historical Serologies for HIV, HBV, HCV, CMV and EBV&	X													
EBV, CMV, PBK DNA PCR+	X				X			X			**X			X
IgG+		X			X			X						X
CRP+		X	X	X	X	X	X	X	**X	**X	**X	**X	**X	X
Donor –specific antibodies (DSA)+	X		X[1]	X[1]	X[1]	X[1]	X[1]	X[1]	**X[1]	**X[1]	**X[1]	**X[1]	**X[1]	X
CNI Levels^	X[4]		X	X	X	X	X	X	**X	**X	**X	**X	**X	X
TB testing [Quantiferon TB Gold]+	X													X
Informed Consent	X													
Inclusion/Exclusion criteria review	X													
Clazakizumab injection		X #1	X #2	X #3	X #4	X #5	X #6	**#7	**#8	**#9	**#10	**#11	**#12	
Anti-clazakizumab (ADA), total IL6, free IL6, and PK(Sent Out)		X			X			X			**X		**X	X
Estimated GFR (using MDRD equation or Schwartz equation will be used to estimate CrCl for patients under 18 years of age)	X		X	X	X	X	X	X						X
Pregnancy test (for WOCP)[2]	X	X	X	X	X	X	X	X	**X	**X	**X	**X	**X	X
Concomitant Med Review	X	X	X	X	X	X	X	X	**X	**X	**X	**X	**X	X
Treg cells (CD4+, CD25+, FoxP3+, CD127+) Tfh cells (CD4+, ICOS+, CXCR5+, IL-21+) Plasmablast (CD19+, CD38+, CD27+), IL-6+) Exosomes Microparticles		X 5mL green top 3mL lavender 2mL blue top			X			X						X
Allograft biopsy	X[3]							X						X**
Review of allograft loss								X						X
Review of non-protocol biopsy results								X						X
Adverse Event Monitoring		X	X	X	X	X	X	X	**X	**X	**X	**X	**X	X

**Based on patient's protocol biopsy results, improvement in GFR, improvement in DSA, and/or PI confirmation, patient will continue another six month course of clazakizumab. If no improvement is observed after 6 months of initial clazakizumab treatment, the patient will come back for last study visit Day 365 after Day 180 protocol biopsy. Allow +/- 30 day for all patient assessments associated with the Day 180 visit

\+ At or within one month prior to screening date & At or within two months prior to screening date ^ At or within 1 month prior to appointment # The biopsy can occur -30 days from day 180 but a minimum of 28 days must elapse between Claz dose #6 and dose #7 1. DSA to be collected prior to clazakizumab infusion. 2. Women of child bearing age is between 12-55 years old. 3. Review of qualifying biopsy for inclusion criteria 4. Review of baseline CNI level 5. Day 365 visit may be combined with the Day 330 visit. All study tests will be obtained but duplicate procedures/tests will be collected once (duplicate items omitted)

Appendix B: Send out labs

Lab	Address
Anti-CLAZ Antibody, PK	ICON Bioanalytical Lab<br>8282 Halsey Road<br>Whitesboro, NY 13492
Donor specific antibody	Cedars-Sinai HLA Laboratory<br>Comprehensive Transplant Center<br>8723 Alden Drive, SSB 197<br>Los Angeles, CA 90048
Transplant Immunology Lab	Cedars-Sinai Transplant Immunology Lab<br>8723 Alden Drive, Steven Spielberg Building, Room 336<br>Los Angeles, CA 90048
Reference Lab	Cedars-Sinai Medical Center Dept of Pathology and Lab Medicine<br>8700 Beverly Blvd, Room 3719<br>Los Angeles, CA 90048

Appendix C: DSMB Charter & FDA Guidance for Clinical Trial Sponsors

FDA Guidance for
Clinical Trial Sponso

DSMB Charter
V2_EC 27Feb2017.dc

Appendix D: Safety Reporting Forms

FDA Med watch
mandatory reporting.

Appendix E Study/Protocol: A Phase I/II Trial to Evaluate the Safety and Tolerability of Clazakizumab® (Anti-IL-6 monoclonal) As an Agent to Eliminate Donor Specific HLA Antibodies (DSAs) and Improve Outcomes of Patients with Chronic & Active Antibody-Mediated Rejection (cABMR) Post-Kidney Transplantation
The below schedule to repeat annually (Visit 1 – 6) per PI discretion

**Study visit**	Visit 1 Follow up year Day 0 CLAZ#1 %	Visit 2 Week 8 -7d/+ 10days	Visit 3 Week 16 -7d/+ 10days	Visit 4 Week 24 -7d/+ 10days	Visit 5 Week 32-7d/+ 10days	Visit 6 Week 40-7d/+ 10days	Day 365 ± 30 days #
Medical History							X#
Complete Physical Exam							X#
Vital signs/weight	X	X	X	X	X	X	
Safety laboratory tests (CBC with differential, CMP, UA)^	X	X	X	X	X	X	
EBV, CMV, PBK DNA PCR^				X			X#
IgG^				X			X#
CRP^				X			X#
Donor –specific antibodies (DSA)^							X#
CNI Levels^	X	X	X	X	X	X	
TB testing [Quantiferon TB Gold]							X&#
Clazakizumab injection	X #1%	X #2	X #3	X #4	X #5	X #6	
Estimated GFR (using MDRD equation or Schwartz equation will be used to estimate CrCl for patients under 18 years of age)	X	X	X	X	X	X	
Pregnancy test (for WOCP)[2]	X	X	X	X	X	X	
Concomitant Med Review	X	X	X	X	X	X	
Kidney Biopsy							X*#
Review of allograft loss							X#
Review of non-protocol biopsy results							X#
Adverse Event Monitoring	X	X	X	X	X	X	

& TB testing to be
completed once a year. TB test will be collected if not done in the last 2 months.
^ At or within 1 month prior to appointment
1. DSA to be collected prior to clazakizumab infusion.
2. Women of child bearing age is between 12-55 years old.
* At 12 Months Per PI discretion (any other biopsies will be conducted for cause)
% Timed minimum 4 weeks from last clazakizumab dose
# Visit should be combined with either visit 1, 2, 3, 5, or 6. Depending on which visit is closest to day 365

Study visit	Visit 1 Follow up year CLAZ#1 %	Visit 2 Week 4 -2d/+ 10days	Visit 3 Week 8 -2d/+ 10days	Visit 4 Week 12 -2d/+ 10days	Visit 5 Week 16 -2d/+ 10days	Visit 6 Week 20 -2d/+ 10days	Day 365 ± 30 days#
Medical History							X#
Complete Physical Exam							X#
Vital signs/weight	X	X	X	X	X	X	
Safety laboratory tests (CBC with differential, CMP, UA)^	X	X	X	X	X	X	
EBV, CMV, PBK DNA PCR^						X	X#
IgG^						X	X#
CRP^						X	X#
Donor –specific antibodies (DSA)^							X#
CNI Levels^	X	X	X	X	X	X	
TB testing [Quantiferon TB Gold]							X &#
Clazakizumab injection	X #1 %	X #2	X #3	X #4	X #5	X #6	
Estimated GFR (using MDRD equation or Schwartz equation will be used to estimate CrCl for patients under 18 years of age)	X	X	X	X	X	X	
Pregnancy test (for WOCP)[2]	X	X	X	X	X	X	
Concomitant Med Review	X	X	X	X	X	X	
Kidney Biopsy							X *#
Review of allograft loss							X#
Review of non-protocol biopsy results							X#
Adverse Event Monitoring	X	X	X	X	X	X	

& TB testing to be completed once a year. TB test will be collected if not done in the last 2 months.

^ At or within 1 month prior to appointment

1. DSA to be collected prior to clazakizumab infusion.
2. Women of child bearing age is between 12-55 years old.

* At 12 Months Per PI discretion (any other biopsies will be conducted for cause)

% Timed minimum 4 weeks from last clazakizumab dose

Visit should be combined with either visit 1, 2, 3, 4 or 5. Depending on which visit is closest to day 365
